# A constrained singular value decomposition method that integrates sparsity and orthogonality

**DOI:** 10.1371/journal.pone.0211463

**Published:** 2019-03-13

**Authors:** Vincent Guillemot, Derek Beaton, Arnaud Gloaguen, Tommy Löfstedt, Brian Levine, Nicolas Raymond, Arthur Tenenhaus, Hervé Abdi

**Affiliations:** 1 Bioinformatics and Biostatistics Hub, Institut Pasteur, Paris, France; 2 The Rotman Research Institute Institution at Baycrest, Toronto, ON, Canada; 3 L2S, UMR CNRS 8506, CNRS–CentraleSupélec–Université Paris-Sud, Université Paris-Saclay, 3 rue Joliot-Curie, 91192 Gif-sur-Yvette, France; 4 Department of Radiation Sciences, Umeå University, Umeå, Sweden; 5 IRMAR, UMR 6625, Université de Rennes, Rennes, France; 6 School of Behavioral and Brain Sciences, The University of Texas at Dallas, Richardson, TX, United States of America; University of Pittsburgh Graduate School of Public Health, UNITED STATES

## Abstract

We propose a new sparsification method for the singular value decomposition—called the constrained singular value decomposition (CSVD)—that can incorporate multiple constraints such as sparsification and orthogonality for the left and right singular vectors. The CSVD can combine different constraints because it implements each constraint as a projection onto a convex set, and because it integrates these constraints as projections onto the intersection of multiple convex sets. We show that, with appropriate sparsification constants, the algorithm is guaranteed to converge to a stable point. We also propose and analyze the convergence of an efficient algorithm for the specific case of the projection onto the balls defined by the norms *L*_1_ and *L*_2_. We illustrate the CSVD and compare it to the standard singular value decomposition and to a non-orthogonal related sparsification method with: 1) a simulated example, 2) a small set of face images (corresponding to a configuration with a number of variables much larger than the number of observations), and 3) a psychometric application with a large number of observations and a small number of variables. The companion R-package, csvd, that implements the algorithms described in this paper, along with reproducible examples, are available for download from https://github.com/vguillemot/csvd.

## Introduction

The singular value decomposition (SVD) [1–3]—the tool “par excellence” of multivariate statistics—constitutes the core of many multivariate methods such as, to name but a few, principal component analysis [4], canonical correlation analysis [5], multiple correspondence analysis [6], and partial least squares methods [7]. To analyze data tables whose rows typically correspond to observations and columns to variables, these statistical methods use the SVD to generate *orthogonal* optimal linear combinations of the variables—called components or factor scores—that extract the most *important* information in the original data. In most cases, only the components that explain the largest proportion of the data variance are kept for further investigation. The coefficients—called loadings—of the linear combination used to compute a component are also often used to understand or “interpret” the corresponding components and this interpretation is greatly facilitated (particularly when the number of variables is large) when, for a given component, only a few variables have large loadings and the other variables have negligible loadings. If this pattern does not naturally hold, several procedures can be used to select the variables that are important for a component. The early psychometric school, for example, would use rotations, such as VARIMAX, [8] of the components in a low dimensional subspace; whereas recent approaches favor computationally based methods such as bootstrap ratios [7], or select important variables using an explicit non-linear optimization method such as the LASSO [9]. Closely related to the current work, in the specific case of principal component analysis (for an extensive review of sparsification for PCA see [10]), Witten et al. (see Section 3.2 in [11]) propose to implement either an orthogonality constraint, or a sparsity constraint (but not both simultaneously, see also, for related ideas, [12, 13]). Along the same lines, Benidis et al. [14] proposed, recently, an algorithm, based on Procrustes approach, for sparse principal component analysis that includes an orthogonality constraint on the loadings.

Unfortunately, in the more general case of having concurrently the sparsity and the orthogonality constraints active on both left and right pseudo-singular vectors, the components obtained from the LASSO and its derivatives are not orthogonal and this often makes their interpretation difficult. To palliate this problem, we present and illustrate a new LASSO-like sparsification method for the SVD, called *constrained singular value decomposition* (CSVD), that incorporates orthogonality constraints on both the rows and the columns of a matrix.

## 1 Notations

Matrices are denoted by uppercase bold letters (e.g., **X**), vectors by lowercase bold (e.g., **x**), and their elements by lower case italic (e.g., *x*_*i*,*j*_). Matrices, vectors, and elements from the same matrix all use the same letter (e.g., **A**, **a**, *a*). The transpose operation is denoted by the superscript “^⊤^”, the inverse of a square matrix **A** is denoted by **A**^−1^. The identity matrix is denoted **I**, vectors or matrices of ones are denoted by **1**, matrices or vectors of zeros are denoted by **0** (when multiplied with or added to other matrices, matrices **I**, **1**, and **0** are assumed to be conformable, in case of doubt their size is specified). When provided with a square matrix, the diag(⋅) operator returns a vector with the diagonal elements of the matrix, and when provided with a vector, the diag(⋅) operator returns a diagonal matrix with the elements of the vector as the diagonal elements of this matrix. When provided with a square matrix, the trace(⋅) operator gives the sum of the diagonal elements of this matrix. The *L*_2_ norm of vector **x**, denoted ‖**x**‖_2_ is defined as ${\left\|x\right\|}_{2}=\sqrt{x^{\;\;⊤}x}$, The *L*_1_ norm of vector **x**, denoted ‖**x**‖_1_ is defined as ‖**x**‖_1_ = ∑(|*x*_*n*_|). A vector **x** is *normalized* by dividing this vector by its *L*_2_ norm (and so a normalized vector has an *L*_2_ norm equal to 1). The Frobenius norm of matrix **X**, denoted ‖**X**‖_*F*_ is defined as ${\left\|X\right\|}_{F}^{2}=\text{trace}\;(X^{\;\;⊤}X)$. The Frobenius inner product of two rectangular matrices **A** and **B** of same dimensions, denoted 〈**A**, **B**〉_*F*_ is defined as ${\langle A,B\rangle }_{F}=\text{trace}\;(AB^{⊤})$. The concatenation of an *I* by *J* matrix **X** and an *I* by 1 vector **y** is the *I* by *J* + 1 matrix denoted [**X**, **y**] obtained by the juxtaposition of **y** on the right side of matrix **X**. The orthogonal complement of the space linearly spanned by the columns of a rectangular matrix **M** is denoted **M**^⊥^. Two rectangular matrices **A** and **B** of same dimensions are said to be orthogonal if and only if 〈**A**, **B**〉_*F*_ = 0.

With *x* being a real scalar and *γ* a non-negative real number, the *scalar soft-thresholding function* denoted *s*(*x*, *γ*) is defined as

$$\begin{array}{c} s\left(x,\gamma \right)=\{\begin{array}{cc} x+\gamma & \;\text{if}\;x<-\gamma ,\\ 0 & \;\text{if}\;|x_{i}|\leq \gamma ,\\ x-\gamma & \;\text{if}\;x>\gamma ; \end{array} \end{array}$$ (1)

and the *vector soft-thresholding function* denoted *S*(**x**, *γ*) is defined as:

$$\begin{array}{c} S\left(x,\gamma \right)=[\begin{array}{c} s(x_{1},\gamma )\\ \vdots \\ s(x_{N},\gamma ) \end{array}]. \end{array}$$ (2)

This function shrinks all the components of **x** toward 0, and set the smallest components to 0.

The projection of a vector **x** onto a space S is denoted by proj(**x**, S). The *L*_2_-ball of radius *ρ*, denoted $B_{L_{2}}\left(\rho \right)$, is defined as

$$\begin{array}{c} B_{L_{2}}\left(\rho \right)=\{x\;|\;\|x{\|}_{2}\leq \rho \} \end{array}$$ (3)

and the *L*_1_-ball of radius *ρ*, denoted denoted $B_{L_{1}}\left(\rho \right)$, is defined as

$$\begin{array}{c} B_{L_{1}}\left(\rho \right)=\{x\;|\;\|x{\|}_{1}\leq \rho \}. \end{array}$$ (4)

Singular values are denoted *δ*, eigenvalues are denoted λ = *δ*^2^.

## 2 Unconstrained singular value decomposition

The SVD of a data matrix $X\in R^{I\times J}$ of rank *L* ≤ min(*I*, *J*) gives the solution of the following problem: Find a least-squares optimal, rank *R* (with *R* ≤ *L*) approximation of **X**, denoted ${\hat{X}}_{\left[R\right]}$. Specifically, the SVD solves the following optimization problem [1, 2, 15]:

$$\begin{array}{c} \underset{{\hat{X}}_{\left[R\right]}\in M_{I,J}\left(R\right)}{\text{arg}\;\text{min}}\frac{1}{2}\|X-{\hat{X}}_{\left[R\right]}{\|}_{F}^{2}=\underset{{\hat{X}}_{\left[R\right]}\in M_{I,J}\left(R\right)}{\text{arg}\;\text{min}}\frac{1}{2}\{\text{trace}({\left(X-{\hat{X}}_{\left[R\right]}\right)}^{\;\;⊤}(X-{\hat{X}}_{\left[R\right]}))\}, \end{array}$$ (5)

where $M_{I,J}\left(R\right)$ is the set of all real *I* × *J* matrices of rank *R*.

Recall that the SVD decomposes **X** as

$$\begin{array}{c} X=P\Delta Q^{\;\;⊤}, \end{array}$$ (6)

where **P**^⊤^**P** = **Q**^⊤^**Q** = **I** and Δ=diag(δ) with *δ*_1_ ≥ *δ*_2_ ≥ ⋯ ≥ *δ*_*L*_ > 0, and *L* is the rank of **X**. The matrix $P\in R^{I\times L}$ (respectively $Q\in R^{J\times L}$) contains the left (respectively right) singular vectors of **X** and the diagonal matrix **Δ** contains the singular values of **X**. If **p**_*ℓ*_ (respectively **q**_*ℓ*_) denotes the *ℓ*-th column of **P** (respectively **Q**), and *δ*_*ℓ*_ the *ℓ*-th element of ***δ***, then, for any *R* ≤ *L*, the optimal matrix ${\hat{X}}_{\left[R\right]}$ is obtained as:

$$\begin{array}{c} {\hat{X}}_{\left[R\right]}=\sum_{\ell =1}^{R}\delta_{\ell }p_{\ell }q_{\ell }^{\;\;⊤} \end{array}$$ (7)

with $p_{\ell }^{\;\;⊤}p_{\ell }=q_{\ell }^{\;\;⊤}q_{\ell }=1$, and $q_{\ell }^{\;\;⊤}q_{\ell^{\prime }}=p_{\ell }^{\;\;⊤}p_{\ell^{\prime }}=0$, ∀ *ℓ* ≠ *ℓ*′.

A classic, albeit non-optimal and potentially numerically unstable, algorithm (described in Algorithm 1) to obtain the unconstrained singular value decomposition of **X** is based on the “power iteration method.” This algorithm—originally developed for the eigen-decomposition of a square matrix—provides the first singular triplet that comprises the first singular value and first left and right singular vectors. In order to ensure orthogonality between successive singular vectors, the first rank one approximation of **X**, computed as

$$\begin{array}{c} {\hat{X}}_{\left[1\right]}=\delta_{1}p_{1}q_{1}^{\;\;⊤}, \end{array}$$ (8)

is subtracted from **X**. This procedure—called deflation (see Appendix A)—gives a new matrix

$$\begin{array}{c} X^{\left(2\right)}=X-\delta_{1}p_{1}q_{1}^{\;\;⊤}, \end{array}$$ (9)

which is orthogonal to ${\hat{X}}_{\left[1\right]}$. The power iteration method is then applied to the deflated matrix **X**^(2)^, giving a second rank one approximation denoted ${\hat{X}}_{\left[3\right]}=\delta_{2}p_{2}q_{2}^{\;\;⊤}$. The deflation is then applied to **X**^(2)^ to give the new residual matrix **X**^(3)^ orthogonal to **X**^(2)^, and so on, until nothing is left to subtract because, then, **X** has been completely decomposed. This way, the optimization problem from Eq 5 can be re-expressed as:

$$\begin{array}{cc} & \underset{\begin{array}{l} \delta_{\ell },p_{\ell },q_{\ell }\\ \ell =1,\ldots ,R \end{array}}{\text{arg}\;\text{min}}\frac{1}{2}\|X-\sum_{\ell =1}^{R}\delta_{\ell }p_{\ell }q_{\ell }^{⊤}{\|}_{F}^{2}\\ & \text{subject}\;\text{to}\;\{\begin{array}{c} p_{\ell }^{⊤}p_{\ell }=1,\\ p_{\ell }^{⊤}p_{\ell^{\prime }}=0,\\ q_{\ell }^{⊤}q_{\ell }=1,\\ q_{\ell }^{⊤}q_{\ell^{\prime }}=0, \end{array}\;\forall \ell^{\prime }\neq \ell . \end{array}$$ (10)

**Algorithm 1**: The power iteration method for the unconstrained SVD. The algorithm consists in alternating the multiplication of the data matrix by the left and right vectors followed by a normalization step. After convergence, the data matrix is deflated and the process is re-iterated.

**Data**: **X**, *ε*, *R*

**Results**: SVD of **X**

Define **X**^(1)^ = **X**;

**for** *ℓ* = 1, …, *R* **do**

 **p**^(0)^ and **q**^(0)^ are randomly initialized;

 *δ*^(0)^ = 0;

 *δ*^(1)^ = **p**^(0)^^⊤^**Xq**^(0)^;

 *s* = 0;

 **while** |*δ*^(*s*+1)^ − *δ*^(*s*)^| ≥ *ε* **do**

  **p**^(*s*+1)^ ← normalize (**Xq**^(*s*)^);

  **q**^(*s*+1)^ ← normalize (**X**^⊤^**p**^(*s*+1)^);

  *δ*^(*s*+1)^ = **p**^(*s*+1)⊤^**Xq**^(*s*+1)^;

  *s* ← *s* + 1;

 **end**

 **X**^(*ℓ*+1)^ = **X**^(*ℓ*)^ − *δ*^(*s*)^ **p**^(*s*)^ **q**^(*s*)⊤^;

**end**

As an alternative to the deflation approach used in Algorithm 1, the orthogonality constraint can be eliminated and integrated into the power iteration algorithm by replacing the normalization steps by the projection of the result of the current iteration onto the intersection of the *L*_2_ ball and the space orthogonal to the previously found left or right singular vectors (see Algorithm 2). This projection onto the intersection of these two spaces can be implemented in a number of ways [16], we chose here to use the projection onto convex sets algorithm (POCS, see, e.g., [17, Page 101])—an iterative algorithm easily implementable and generalizable to the projection onto the intersection of more than two convex sets (see Algorithm 3). Recall that, when normalized, a vector **x** is projected onto the *L*_2_ ball and that a vector **x** is projected onto **V**^⊥^ by multiplying it by **I** − **V**^⊤^ **V**. In POCS, these two projection steps are iterated until convergence.

**Algorithm 2** An alternative algorithm of the power iteration for the unconstrained SVD: The deflation step is replaced by a projection onto the space orthogonal to the space defined by the already computed lower rank version of the data matrix. Note that **0**^⊥^ corresponds to the whole space, so it is either $R^{I}$ or $R^{J}$.

**Data**: **X**, *ε*, *R*

**Result**: SVD of **X**

Define **P** = **0**;

Define **Q** = **0**;

**for** *ℓ* = 1, …, *R* **do**

 **p**^(0)^ and **q**^(0)^ are randomly initialized;

 *δ*^(0)^ ← 0;

 *δ*^(1)^ ← **p**^(0)⊤^**Xq**^(0)^;

 *s* ← 0;

 **while** |*δ*^(*s*+1)^ − *δ*^(*s*)^| ≥ *ε* **do**

  **p**^(*s*+1)^ ← proj(**Xq**^(*s*)^, $B_{L_{2}}\left(1\right)\cap P^{⊥}$);

  **q**^(*s*+1)^ ← proj(**X**^⊤^ **p**^(*s*+1)^, $B_{L_{2}}\left(1\right)\cap Q^{⊥}$);

  *δ*^(*s*+1)^ ← **p**^(*s*+1)⊤^ **Xq**^(*s*+1)^;

  *s* ← *s*+1;

 **end**

 *δ*_*ℓ*_ ← *δ*^(*s*+1)^;

 **P** ← [**P**, **p**^(*s*+1)^];

 **Q** ← [**Q**, **q**^(*s*+1)^];

**end**

**Algorithm 3** Projection onto the intersection of *K* convex sets (POCS).

**Data**: **x**, $S_{1},\ldots ,S_{K}$, *ε*

**Results**: Projection of **x** onto $\overset{K}{\underset{k=1}{⋂}}S_{k}$

Define **x**^(0)^ = **x**;

**while** |**x**^(*s*+1)^ − **x**^(*s*)^| ≥ *ε* **do**

 **x**^(*s*+1)^ ← proj(**x**^(*s*)^, $S_{1}$));

 **for** *k* = 2, …, *K* **do**

  **x**^(*s*+1)^ ← proj(**x**^(*s*+1)^, $S_{k}$));

 **end**

 *s* ← *s* + 1

**end**

Algorithm 1 is obviously faster than Algorithm 2 as implemented using Algorithm 3, because the orthogonality constraint in Algorithm 1 is performed with one operation whereas Algorithm 2 always requires several operations. However, the main benefit of Algorithm 2 is that it easily can be extended to include additional constraints, as illustrated below.

## 3 Constrained singular value decomposition

### 3.1 Previous work

**Algorithm 4**: The Algorithm of Witten et al. [11]: The penalized matrix decomposition (PMD) approach.

**Data**: **X**, *ε*, *R*

**Resulet**: SVD of **X**

Define **X**^(1)^ = **X**;

**for** *ℓ* = 1, …, *R* **do**

 **p**^(0)^ and **q**^(0)^ are randomly initialized;

 *δ*^(0)^ = 0;

 *δ*^(1)^ = **p**^(0)⊤^**Xq**^(0)^;

 *s* = 0;

 **while** |*δ*^(*s*+1)^ − *δ*^(*s*)^| ≥ *ε* **do**

  **p**^(*s*+1)^ ← normalize (*S*(**Xq**^(*s*)^, λ_1,*ℓ*_)), with λ_1,*ℓ*_ such that ‖**p**^(*s*+1)^‖_1_ = *c*_1,*ℓ*_;

  **q**^(*s*+1)^ ← normalize (*S*(**X**^⊤^ **p**^(*s*+1)^, λ_2,*ℓ*_)), with λ_2,*ℓ*_ such that ‖**q**^(*s*+1)^‖_1_ = *c*_2,*ℓ*_;

  *δ*^(*s*+1)^ = **p**^(*s*+1)⊤^ **Xq**^(*s*+1)^;

  *s* ← *s* + 1;

 **end**

 **X**^(*ℓ*+1)^ = **X**^(*ℓ*)^ − *δ*^(*s*)^ **p**^(*s*)^ **q**^(*s*)⊤^;

**end**

Recently, several authors have proposed sparse variants of the SVD (see e.g., [4, 11, 15, 18, 19] for reviews), or, more specifically, of PCA [14, 20]. For most of these sparse variants, the sparsification is obtained by adding new constraints to Eq 10. For example, the penalized matrix decomposition (PMD) approach by Witten et al. [11] solves the following optimization problem for the first pair of left and right singular vectors:

$$\begin{array}{l} \underset{\begin{array}{l} \delta_{\ell },p_{\ell },q_{\ell }\\ \ell =1,\ldots ,R \end{array}}{\text{arg}\;\text{min}}\frac{1}{2}\|X-\sum_{\ell =1}^{R}\delta_{\ell }p_{\ell }q_{\ell }^{⊤}{\|}_{F}^{2}\\ \text{subject}\;\text{to}\;\{\begin{array}{l} p_{\ell }^{⊤}p_{\ell }=1,\\ q_{\ell }^{⊤}q_{\ell }=1,\\ C_{1}\left(p_{\ell }\right)\leq c_{1,\ell },\\ C_{2}\left(q_{\ell }\right)\leq c_{2,\ell }, \end{array} \end{array}$$ (11)

where *C*_1_ and *C*_2_ are convex penalty functions from $R^{I}$ (respectively $R^{J}$) to $R^{+}$ (such as, e.g., the LASSO, or the fused LASSO constraints) and with *c*_1,*ℓ*_ and *c*_2,*ℓ*_ being positive constants. The PMD procedure is described in Algorithm 4. In PMD, the next pseudo-singular triplet is estimated by solving the same optimization problem where **X** is replaced by a deflated matrix. In contrast to Eq 11, however, the added constraints create a nonlinear optimization problem and this makes the deflated matrices non-orthogonal to the previous rank one optimal matrix [21]. This lack of orthogonality makes the interpretation of the components somewhat difficult because the conclusions about one component involve all correlated components and because the same information is explained (to different degrees) by all correlated components. In the specific case of PCA, Witten et al. proposed, alternatively, to impose an orthogonality constraint on the left singular vectors, without the sparsity constraint, and to leave the sparsity constraint active only on the right vectors (i.e., the loadings). However, this procedure does not solve the problem of having both constraints simultaneously active on the left and right singular vectors (See Equation 3.17 and the subsequent algorithm in [11] for more details).

In order to eliminate the problems created by the non-orthogonality of the singular vectors, we present below a new optimal sparsification method, called the *constrained singular value decomposition* (CSVD) that implements orthogonality constraints on successive sparsified singular vectors.

### 3.2 Current work: The constrained SVD (CSVD)

The constrained SVD still decomposes the matrix **X** into singular values and vectors, but imposes, in addition, on the singular vectors constraints that induce sparsity of the weights. Such sparsity-inducing constraints are common in fields where the data comprise large numbers of variables [22] (e.g., tens of thousands, as in genomics [23], to millions, as in neuroimaging [24, 25]). Although the theory of sparsity-inducing constraints is well documented, we are interested in a general formulation that could also be applied for several types of sparsification, as well as more sophisticated constraints.

Specifically, we consider the following optimization problem:

$$\begin{array}{cc} & \underset{\begin{array}{l} \delta_{\ell },p_{\ell },q_{\ell }\\ \ell =1,\ldots ,R \end{array}}{\text{arg}\;\text{min}}\frac{1}{2}\|X-\sum_{\ell }^{R}\delta_{\ell }p_{\ell }q_{\ell }^{⊤}{\|}_{F}^{2}\\ & \text{subject}\;\text{to}\;\{\begin{array}{c} p_{\ell }^{⊤}p_{\ell }=1,\\ p_{\ell }^{⊤}p_{\ell^{\prime }}=0,\\ q_{\ell }^{⊤}q_{\ell }=1,\\ q_{\ell }^{⊤}q_{\ell^{\prime }}=0, \end{array}\;\forall \ell^{\prime }\neq \ell ,\\ & \text{and}\;\text{to}\;\{\begin{array}{c} C_{1}\left(p_{\ell }\right)\leq c_{1,\ell },\\ C_{2}\left(q_{\ell }\right)\leq c_{2,\ell }, \end{array} \end{array}$$ (12)

where *C*_1_ and *C*_2_ are convex penalty functions from $R^{I}$ (respectively $R^{J}$) to $R^{+}$, (which could be, e.g., the LASSO, group-LASSO, or fused LASSO constraints) and with *c*_1,*ℓ*_ and *c*_2,*ℓ*_ being positive constants: smaller values of *c*_1,*ℓ*_ and *c*_2,*ℓ*_ lead to solutions that are more sparse. See, however, e.g., [11, 26], and, as developed in Appendix B, only some ranges of values of *c*_1,*ℓ*_ and *c*_2,*ℓ*_ will lead to solutions.

An equivalent, but more convenient, form of the problem described in Eq 12 can be derived by considering two orthogonal matrices **P**, and **Q**, and a diagonal matrix **Δ** = diag{***δ***} such that

$$\begin{array}{c} \frac{1}{2}\|X-P\Delta Q^{\;\;⊤}{\|}_{F}^{2}=\frac{1}{2}\|X{\|}_{F}^{2}+\frac{1}{2}\sum_{\ell }\delta_{\ell }^{2}-\sum_{\ell }\delta_{\ell }p_{\ell }^{\;\;⊤}Xq_{\ell }\;. \end{array}$$ (13)

The term $\|X{\|}_{F}^{2}$ is constant and $\sum \delta_{\ell }^{2}$ does not depend on **p**_*ℓ*_ or **q**_*ℓ*_, and so for a given *δ*_*ℓ*_ positive, the solutions of $\arg\max\delta_{\ell }p_{\ell }^{⊤}Xq_{\ell }$ are the same as the solutions of $\arg\max p_{\ell }^{⊤}Xq_{\ell }$. In addition the maximum is reached when $\delta_{\ell }=p_{\ell }^{⊤}Xq_{\ell }$ (see, e.g., [11]). Consequently, minimizing $\|X-P\Delta Q^{\;\;⊤}{\|}_{F}^{2}$ from Eq 13 is equivalent to maximizing each term of the sum $\sum p_{\ell }^{\;\;⊤}Xq_{\ell }$. Therefore, Eq 12 is equivalent to the following 1-dimensional maximization problem for *ℓ* ≥ 1, given the previous vectors **p**_*ℓ*′_ and **q**_*ℓ*′_ for all 0 ≤ *ℓ*′ < *ℓ*:

$$\begin{array}{cc} & \underset{p,q}{arg max}\{p^{\;\;⊤}Xq\}\\ & \text{subject}\;\text{to}\;\{\begin{array}{c} p^{\;\;⊤}p=1,\\ q^{\;\;⊤}q=1, \end{array}\;\text{and}\;\forall \;\ell^{\prime }<\ell \{\begin{array}{c} p^{\;\;⊤}p_{\ell^{\prime }}=0,\\ q^{\;\;⊤}q_{\ell^{\prime }}=0, \end{array}\;\text{and}\;\{\begin{array}{c} C_{1}(p)\leq c_{1,\ell },\\ C_{2}(q)\leq c_{2,\ell }, \end{array} \end{array}$$ (14)

where we set (for convenience) **p**_0_ and **q**_0_ to **0** (note that for the first pseudo-singular vectors, the orthogonality constraint is not active because all vectors are orthogonal to **0**).

To facilitate the resolution of this optimization problem, the unicity constraint on the *L*_2_ norm of the singular vectors (i.e., **p**^⊤^**p** = 1 and **q**^⊤^**q** = 1) needs to be relaxed and replaced by an equivalent inequality. Specifically, the optimization problem in Eq 14 is reframed as

$$\begin{array}{cc} & \underset{p,q}{arg max}\{p^{\;\;⊤}Xq\}\\ & \text{subject}\;\text{to}\;\{\begin{array}{c} p^{\;\;⊤}p\leq 1,\\ q^{\;\;⊤}q\leq 1, \end{array}\text{and}\;\;\forall \;\ell^{\prime }<\ell \{\begin{array}{c} p^{\;\;⊤}p_{\ell^{\prime }}=0,\\ q^{\;\;⊤}q_{\ell^{\prime }}=0, \end{array}\text{and}\;\;\{\begin{array}{c} C_{1}(p)\leq c_{1,\ell },\\ C_{2}(q)\leq c_{2,\ell }. \end{array} \end{array}$$ (15)

Eq 15 defines a biconcave maximization problem with convex constraints. This problem can be solved using block relaxation [27]: An iterative algorithm that consists in a series of two-part iterations in which (Part 1) the expression in Eq 15 is maximized for **p** with **q** being fixed, and is then (Part 2) maximized for **q** with **p** being fixed. Part 1 of the iteration can be re-expressed as the following optimization problem:

$$\begin{array}{cc} & \underset{p}{arg min}\{\frac{1}{2}\|p-Xq{\|}_{2}^{2}\}\\ & \text{subject to}\{\begin{array}{c} p\in B_{L_{2}}\left(1\right),\\ p\in B_{L_{1}}\left(c_{1}\right),\\ p\in P^{⊥}. \end{array} \end{array}$$ (16)

Eq 16 shows that finding the optimal value for **p** (i.e., Part 1 of the alternating procedure) is equivalent to finding the projection of the vector **Xq** onto the subspace of $R^{I}$ defined by the intersection of all the convex spaces involved by the constraints. During Part 2 of the alternating procedure, the vector **p** is fixed and therefore Part 2 can be expressed as the following minimization problem:

$$\begin{array}{cc} & \underset{q}{arg min}\{\frac{1}{2}\|q-X^{⊤}p{\|}_{2}^{2}\}\\ & \text{subject to}\{\begin{array}{c} q\in B_{L_{2}}\left(1\right),\\ q\in B_{L_{1}}\left(c_{2}\right),\\ q\in Q^{⊥}. \end{array} \end{array}$$ (17)

Eqs 16 and 17 replace the *L*_1_ and *L*_2_ constraints in the minimization problem expressed in Eq 11 by projections onto the intersection of the convex sets (POCS) defined by these constraints. Because the intersection of several convex sets is also a convex set [28], the block relaxation algorithm from Eq 15 is essentially composed of sequential series of operations applied until convergence of the two projections onto their respective convex sets. This strategy can obviously be extended to incorporate multiple additional constraints as long as these constraints define convex subspaces. As shown in Appendix D, the CSVD algorithm is guaranteed to converge to a stable point because it is a member of the more general class of the *block successive upper-bound minimization (BSUM) algorithms*.

In the specific case of the projection on the intersection of the balls *L*_1_ and *L*_2_, POCS can be replaced by a fast and exact algorithm called PL1L2 ([26], see Appendix C for details). This algorithm (see Algorithm 5) differs from the more general Algorithm 4 only by the specification of the projection method onto the *L*_1_ ball which is implemented as a simple and fast algorithm based on the soft-thresholding operator.

Note that, Algorithm 2—presented in Section 2 for the unconstrained SVD and using POCS for the projection onto an intersection of convex sets—can be easily generalized to incorporate a new sparsity constraint, by simply applying POCS to the intersection of 3 convex sets (instead of just 2 convex sets): the *L*_2_-ball of radius 1, an *L*_1_-ball, and the orthogonal subspace to the space defined by the previously found left or right pseudo-singular vectors.

**Algorithm 5**: General algorithm for the Constrained Singular Value Decomposition. The projection onto the *L*_1_-ball can be replaced by another projection onto a convex set, making it possible to adapt this algorithm to other purposes.

**Data**: **X**, *ε*, *R*, *c*_1,*ℓ*_ and *c*_2,*ℓ*_ for *ℓ* in 1, …, *R*

**Results**: CSVD of **X**

Define **P** = **0**;

Define **Q** = **0**;

**for** *ℓ* = 1, …, *R* **do**

 **p**^(0)^ and **q**^(0)^ are randomly initialized;

 *δ*^(0)^ ← 0;

 *δ*^(1)^ ← **p**^(0)⊤^**Xq**^(0)^;

 *s* ← 0;

 **while** |*δ*^(*s*+1)^ − *δ*^(*s*)^| ≥ *ε* **do**

  **p**^(*s*+1)^ ← proj(**Xq**^(*s*)^, $B_{1}(c_{1,\ell })\cap B_{2}(1)\cap P^{⊥}$);

  **q**^(*s*+1)^ ← proj(**X**^⊤^**p**^(*s*+1)^, $B_{1}(c_{2,\ell })\cap B_{2}(1)\cap Q^{⊥}$);

  *δ*^(*s*+1)^ ← **p**^(*s*+1)⊤^**Xq**^(*s*+1)^;

  *s* ← *s* + 1;

 **end**

 *δ*_*ℓ*_ ← *δ*^(*s*+1)^;

 **P** ← [**P**, **p**^(*s*+1)^];

 **Q** ← [**Q**, **q**^(*s*+1)^];

**end**

In the following sections, we illustrate—using simulated and real data—the effect and importance of the orthogonality constraint and show how this constraint improves the interpretability of the analysis.

## 4 Empirical comparative evaluation of the CSVD

In this section, we empirically evaluate, illustrate the constrained singular value decomposition (CSVD), and compare its performance to the performance of the plain SVD and the closely related sparsification method of Witten et al. [11], the PMD method. To do so, we used: 1) some simulated datasets, 2) one simulated dataset mimicking a real dataset, and 3) one real dataset (the characteristics of these datasets are listed in Table 1).

**Table 1:** Characteristics of the various datasets used to assess the performance of the CSVD and related methods.

Dataset	*I* (# of rows)	*J* (# of columns)	Rank
Simulated	150	600	149
Face Data	6	55,200	6
Memory	2,100	30	30

With the first (simulated) dataset we evaluated how the SVD, PMD, and CSVD recover the ground truth for a relatively large dataset with more variables than observations contains a mixture of signal and Gaussian noise.

Datasets two and three were chosen to each illustrate a particular aspect of the data. The second dataset investigates the *N* ≪ *P* problem and comprises six face images consisting of 230 × 240 = 55, 200 pixels—with each pixel measuring light intensity on a scale going from 0 to 255. The third dataset illustrates the effects of sparsification on a dataset corresponding to a traditional psychometric problem. This simulated has been created to match the pattern of loadings of a real dataset that was collected from 2, 100 participants who—as part of a larger project on memory—answered an online version of the “object-spatial imagery questionnaire” (OSIQ, [29])—a psychometric instrument measuring mental imagery for objects and places. Using a 5-point rating scale, participants rated their agreement for 30 items that should span a 2-dimensional space corresponding to the spatial and object imagery psychometric factors. This dataset is used to compare sparsification and the standard traditional psychometric approach relying on (Varimax) rotation to recover a two dimensional factor structure.

For Datasets two and three, we applied three degrees of sparsity (low, medium, and high). As detailed in Appendix B, only some values of *c*_1_ and *c*_2_ will lead to solutions (specifically, *c*_1_ has to be chosen between 1 and $\sqrt{I}$ and *c*_2_ between 1 and $\sqrt{J}$). Table 2 lists the values chosen for *c*_1_ and *c*_2_ and their interpretation for PMD and the CSVD. Also, for technical reasons, the values of *c*_1_ and *c*_2_ corresponding to the maximum sparsity for **P** and **Q** are set, respectively, to 1 + *ε*_1_ and 1 + *ε*_2_ (instead of 1) with *ε*_1_ and *ε*_2_ being two small real positive values.

**Table 2:** The different values of the sparsity parameters for the CSVD and PMD.

*c*_1_	*c*_2_	Resulting degree of sparsity	Notation
1 + *ε*_1_	1 + *ε*_2_	The sparsest level, most of the coefficients are close zero	H (High)
$\frac{1}{3}\sqrt{I}$	$\frac{1}{3}\sqrt{J}$	Very sparse	M (Medium)
$\frac{2}{3}\sqrt{I}$	$\frac{2}{3}\sqrt{J}$	Somewhat sparse	L (Low)
$\sqrt{I}$	$\sqrt{J}$	No sparsity, corresponds to the regular SVD	N (None)

### 4.1 Simulated data

With these simulated data, we evaluate the ability of the CSVD to recover known singular triplets, their sparsity structure, and the orthogonality of the estimated left and right singular vectors. These simulated data were created by adding a matrix of Gaussian noise to a 150 by 600 matrix of rank 5 built from its SVD decomposition.

Specifically, the data matrix **X** was created as

$$\begin{array}{c} X=X_{M}+E, \end{array}$$ (18)

where

- $X_{M}=P_{M}\Delta_{M}Q_{M}^{⊤}$ is the rank 5 matrix of the ground truth with:
  - **Δ**_*M*_ a 5 × 5 the diagonal matrix of the singular values equal to **Δ**_*M*_ = diag (***δ***) = diag([15, 14, 13, 12, 11])
  - **P**_*M*_ an *I* = 150 × 5 = 750 by 5, orthogonal matrix with **P**^⊤^**P** = **I**,
  - **Q**_*M*_ a *J* = 600 × 5 = 3, 000 by 5 orthogonal matrix with **Q**^⊤^**Q** = **I**,
- **E** is an *I* = 150 × *J* = 600 matrix containing *I* × *J* = 90, 000 independent realizations of a Gaussian variable with mean equal to 0 and standard deviation equal to 0.01.

Matrices **P**_*M*_ and **Q**_*M*_ were designed to be both sparse and orthogonal. Specifically, matrix **P**_*M*_ was generated with the following model

$$\begin{array}{c} P_{M}=[\begin{array}{ccccc} p_{1} & p_{2} & p_{3} & p_{4} & p_{5}\\ p_{1}^{\prime } & 0 & 0 & 0 & 0\\ 0 & p_{2}^{\prime } & 0 & 0 & 0\\ 0 & 0 & p_{3}^{\prime } & 0 & 0\\ 0 & 0 & 0 & p_{4}^{\prime } & 0\\ 0 & 0 & 0 & 0 & p_{5}^{\prime } \end{array}], \end{array}$$ (19)

where **0** denotes 25 × 1 vectors of 0s, and where all **p** and **p**′ were 25 × 1 vectors with norm equal to $2^{-\frac{1}{2}}$ and such that where $p_{\ell }^{\;\;⊤}p_{\ell^{\prime }}=0,\forall \;\ell \neq \ell^{\prime }$. A similar model was used for **Q**_*M*_ which was generated with the following model

$$\begin{array}{c} Q_{M}=[\begin{array}{ccccc} q_{1} & q_{2} & q_{3} & q_{4} & q_{5}\\ q_{1}^{\prime } & 0 & 0 & 0 & 0\\ 0 & q_{2}^{\prime } & 0 & 0 & 0\\ 0 & 0 & q_{3}^{\prime } & 0 & 0\\ 0 & 0 & 0 & q_{4}^{\prime } & 0\\ 0 & 0 & 0 & 0 & q_{5}^{\prime } \end{array}], \end{array}$$ (20)

where **0** were 120 × 1 vectors of 0s, and where all **q q**′ vectors were 120 × 1 vectors with norm equal to $2^{-\frac{1}{2}}$ and such that $q_{\ell }^{\;\;⊤}q_{\ell^{\prime }}=0,\forall \;\ell \neq \ell^{\prime }$.

With the structure described in Eqs 19 and 20, the low-rank matrix to recover from **X** is then composed of 2 parts: 1) a common part (no sparsity) to all 5 components (i.e., the part corresponding to the **p** and **q** vectors), and 2) one part specific to each component (i.e., the **p**′ and the **q**′ vectors) with the corresponding part in the other 4 components being sparse.

**Fig 1:**
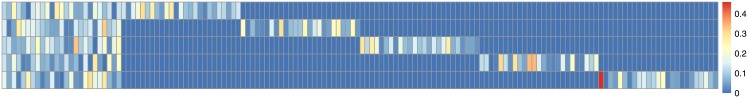
Simulated data. Heatmap of $P_{M}^{⊤}$: the *true* left singular vectors (in an horizontal representation: one line equals one singular vector).

**Fig 2:**
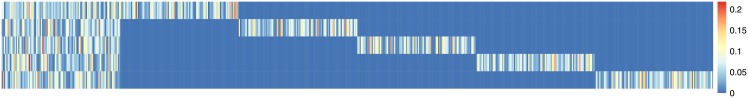
Simulated data. Heatmap of $Q_{M}^{⊤}$: the *true* right singular vectors (in an horizontal representation: one line equals one singular vector).

Figs 1 and 2 show heatmaps of the true left-singular vectors (**P**_*M*_) and right-singular vectors (**Q**_*M*_).

We analyzed **X** with the CSVD and PMD. For both methods, the *L*_1_ constraint was set to *c*_1_ = 5 for the left-singular vectors and *c*_2_ = 11 for the right-singular vectors, based on the sparsity of the ground truth.

**Fig 3:**
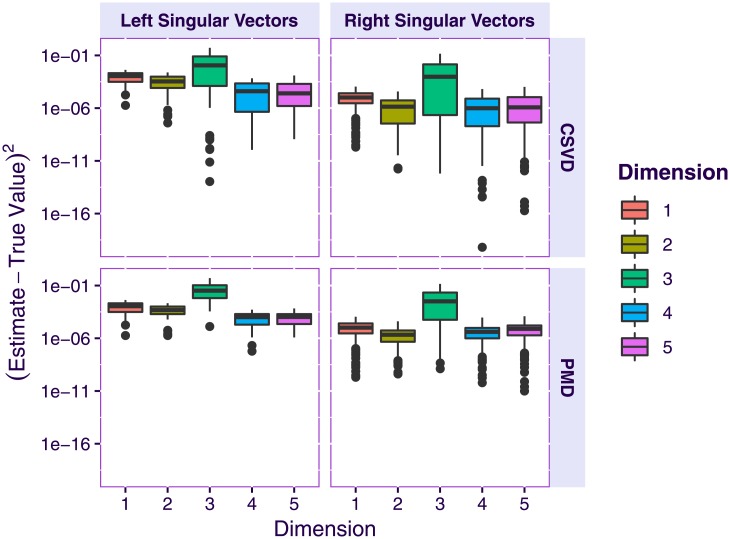
Simulated data. Boxplots of the squared difference between the estimated singular vectors and the ground truth.

**Fig 4:**
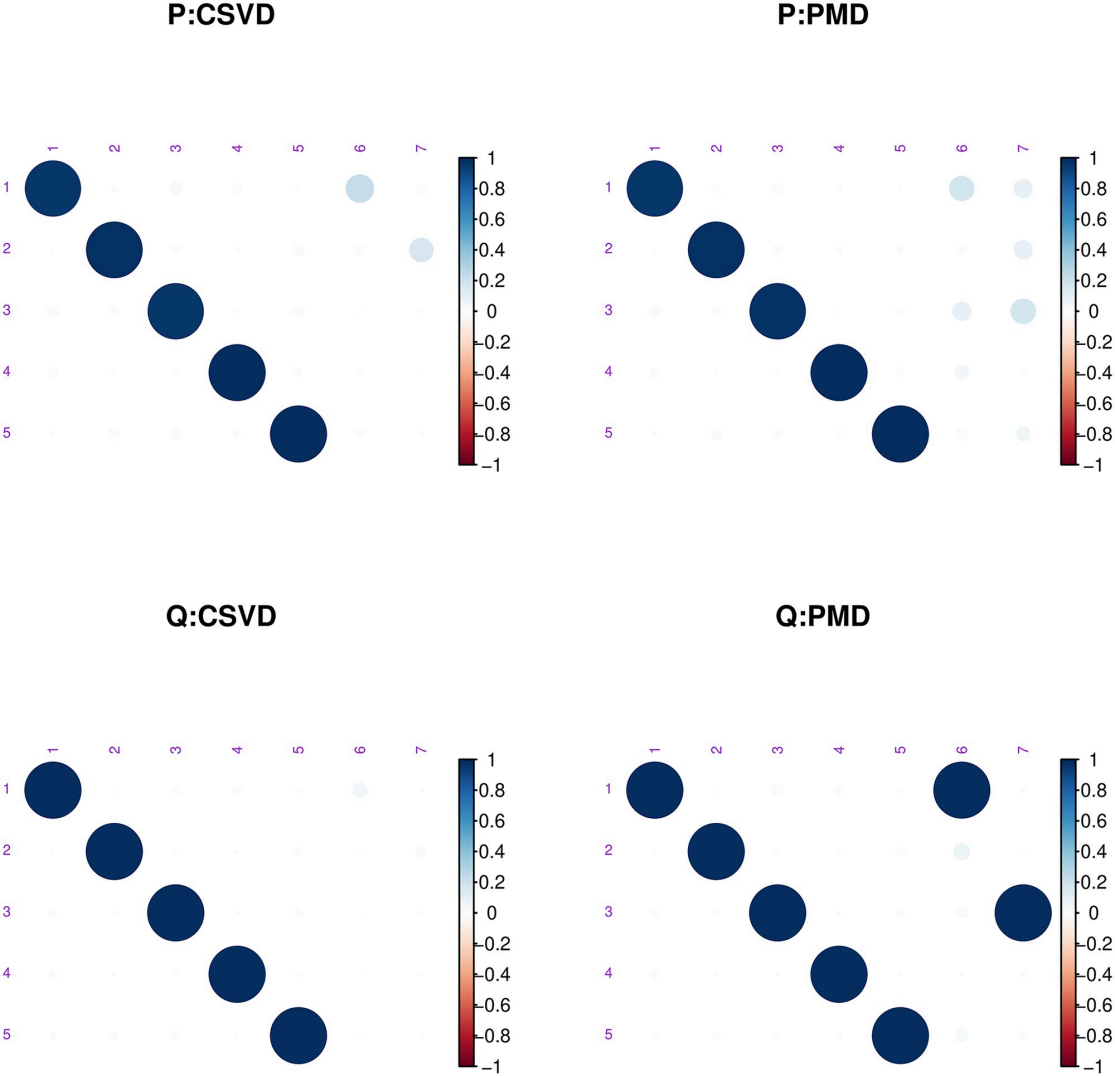
Simulated data. Heatmap of the correlations between the estimated left (**P**) and right (**Q**) singular vectors with the ground truth for the CSVD and PMD. Each cell of the heatmap represents the correlation between one of the 7 estimated (left or right) vectors with the 5 true vectors. Each heatmap contains 5 rows (the ground truth) and 7 columns (the estimated vectors). On the left, are the results obtained with the CSVD and on the right, the results obtained with PMD.

We asked each method to return 7 vectors in order to evaluate if the methods could recover the ground truth (i.e., the 5-dimensional sub-space) but also how they would behave after this 5-dimensional subspace had been recovered. Fig 3 shows the boxplots of the distribution of the squared difference between the estimated singular vectors and the ground truth for PMD and the CSVD: Both methods correctly uncover the singular vectors. Fig 4 shows the correlations between the first 7 estimated singular vectors compared to the ground truth: Although the first 5 singular vectors are correctly estimated, and, roughly, orthogonal to the previous singular vectors, the 2 last vectors estimated by PMD are correlated to some of the previously computed vectors. This demonstrates the failure of the standard deflation technique to impose the orthogonality constraint when a non-linear optimization methods is used. By contrast with the PMD approach, the orthogonality constraint of the CSVD prevented this problem.

**Fig 5:**
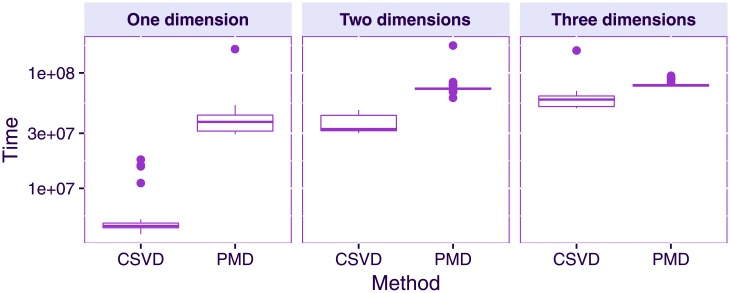
Simulated data. Computational time of PMD and the CSVD when estimating one sparse singular triplet (left) and two sparse singular triplets (right).

Fig 5 displays the computational times of the CSVD and PMD as a function of the number of computed components: the CSVD is faster than PMD for the estimation of the first component, but this advantage diminishes when the number of computed components increases. This pattern indicates that the orthogonality constraint increases the computational time.

Table 3 contains the estimated singular values for the CSVD and PMD as well as the ground-truth singular values. The estimated pseudo-singular values are comparable for both methods and behave in a similar way compared to the ground truth and to the regular SVD: The first singular values are slightly smaller than their ground truth values, but at some point—which varies depending on the imposed degree of sparsity—the estimated number of pseudo-singular values is larger than the ground truth.

**Table 3:** Estimated singular values and ground truth. In the “ground truth” column, a value of 0 indicates that the corresponding real singular value does not exist (i.e., because the underlying matrix has rank 5).

Order	CSVD	PMD	SVD	Ground Truth
1	14.77	14.77	14.97	15.00
2	13.62	13.64	13.99	14.00
3	12.53	12.59	12.99	13.00
4	11.86	11.90	11.97	12.00
5	10.34	10.45	10.93	11.00
6	0.21	2.94	0.04	0
7	0.15	2.98	0.04	0

Additionally, broader settings were considered for the comparison of SVD, CSVD and PMD on simulated data. Specifically, we considered a case with a low signal to noise ratio, and a case where the noise is structured: PMD and CSVD performed equally poorly on noisy data, but were unaffected by a structured noise. These additional results are reported in S1 Table.

To sum up: 1) the CSVD and PMD produce highly similar estimates of the first singular vectors; 2) the CSVD and PMD both recover the true sparsity structure of the ground-truth data; 3) for singular vectors of an order higher than the rank of the matrix, PMD produces singular vectors correlated with the previous ones; and 4) the CSVD is computationally more efficient than PMD but this advantage shrinks as the number of computed components increases.

### 4.2 The face data

The dataset consists in six 240 × 230 = 55, 200 gray scale digitized (range from 0 to 255) face images (three men and three women) that were extracted from a larger face database (see [30, 31]) and are available as the dataset sixFaces from the R-package data4PCCAR (obtained from the Github repository HerveAbdi/data4PCCAR).

**Fig 6:**
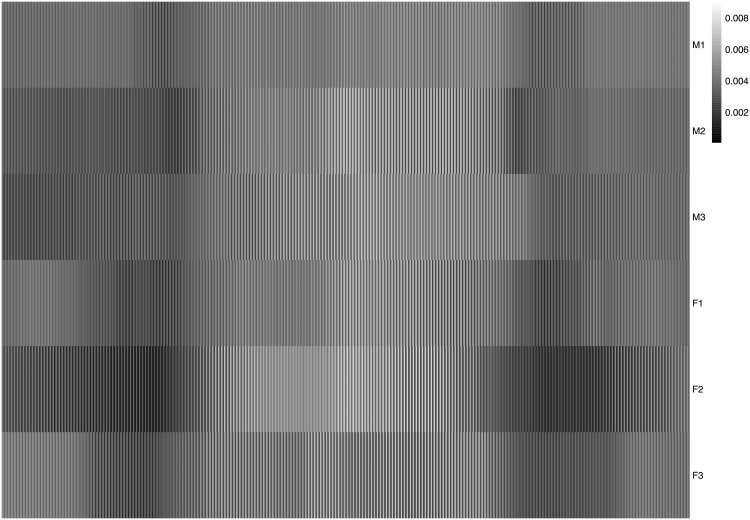
Face data. The data matrix of the face dataset: 6 faces by 55,200 voxels. The female faces are denoted F1, F2, and F3, the male faces M1, M2, and M3.

**Fig 7:**
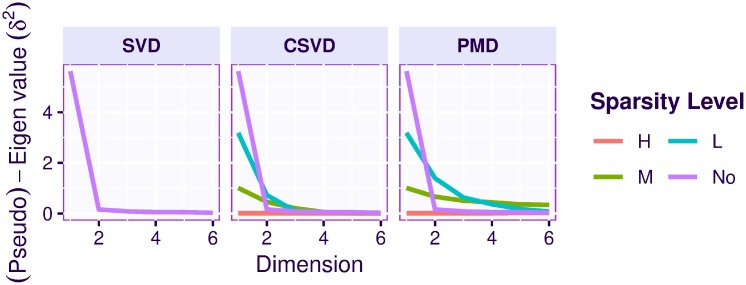
Face data. Eigenvalues and pseudo-eigenvalues per dimension. Left column: eigenvalues obtained from regular SVD; middle column: pseudo-eigenvalues (i.e., variance of the factor scores) for the CSVD; right column: pseudo-eigenvalues (i.e., variance of the factor scores) for the PMD. For PMD and the CSVD, each line represents a level of sparsity: none (No), low (L), medium (M), and high (H).

**Fig 8:**
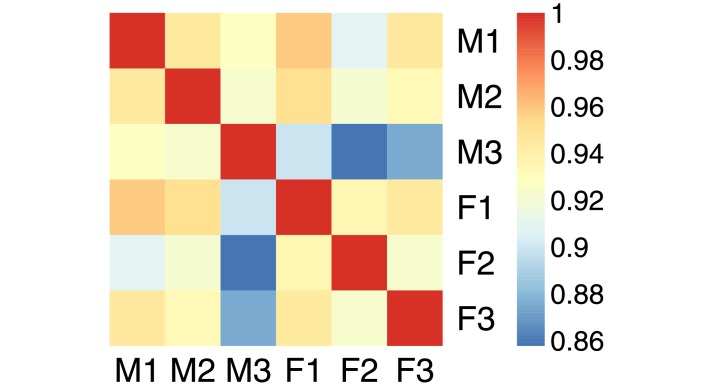
Face data. Cosine matrix for the 6 faces of the face dataset. The female faces are denoted F1, F2, and F3, the male faces M1, M2, and M3.

**Fig 9:**
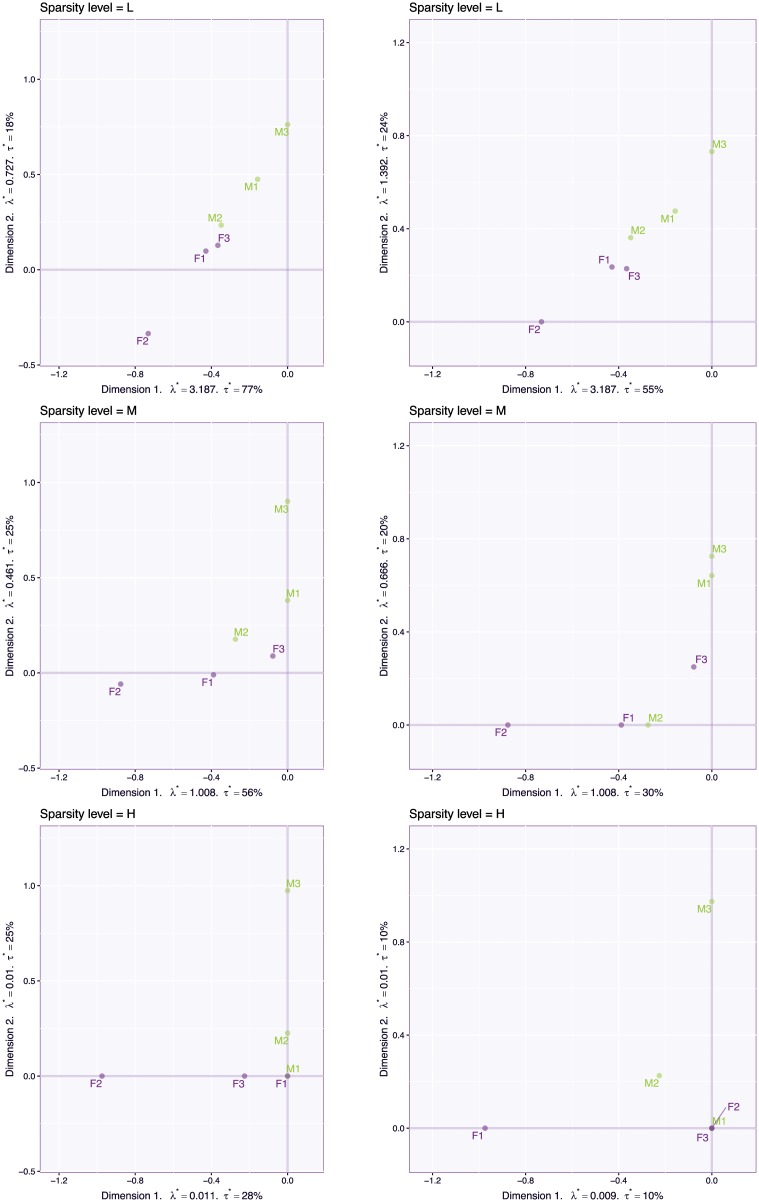
Face data. First two sparse left singular vectors (**P**) for the CSVD (left) and PMD (right) for three levels of sparsity: low (L), medium (M), and high (H). The results for the CSVD are reported on the left, and the results for PMD are reported on the right.

Each image was unfolded (i.e., “vectorized”) into a 240 × 230 = 55, 200 element vector, which was re-scaled to norm one. A plain SVD was then performed on the 6 × 55, 200 matrix (see Fig 6) obtained from the concatenation of the 6 face vectors. The SVD extracted 6 components with the first one extracting a very large proportion of the total variance (i.e., λ_1_ = 5.616, *τ*_1_ = 94%, see also Fig 7 left panel). This very large first eigenvalue indicates that these face images are highly correlated (see Fig 8)—An interpretation confirmed by the very similar values of the coordinates of the six faces for the first component. But this large first eigenvalue reflects also, in part, that the data were not centered, because, with all entries of the matrix being positive, the first left (respectively right) singular vector (i.e., the first component) is respectively, the 6-element long vector of a weighted mean of the pixels across the faces (respectively the 55, 200-element long vector of a weighted mean of the faces across the pixels) and so all elements of the first pair of singular vectors have the same sign, see Table 4 and the picture of the first “eigen-face” [32] in the left of the top row of Fig 9). The second component differentiates females and males (see the map of the faces for Components 1 versus 2 in Fig 9, and the picture of the second “eigen-face” in the right of the top row of Fig 9).

**Table 4:** Face example. Left singular vectors (i.e., face loadings) and associated eigenvalues.

Dimension	Faces						Eigenvalue	Percentage
	1	2	3	4	5	6	λ	*τ*
1	−.41	−.41	−.40	−.41	−.40	−.41	5.616	93.61
2	.14	.09	.76	−.16	−.53	−.30	0.160	2.66
3	−.40	.13	.29	−.14	.67	−.52	0.086	1.43
4	.08	−.68	.34	−.41	.27	.42	0.055	0.91
5	.45	−.54	−.04	.50	.11	−.50	0.052	0.87
6	−.66	−.25	.25	.60	−.16	.22	0.031	0.52

**Fig 10:**
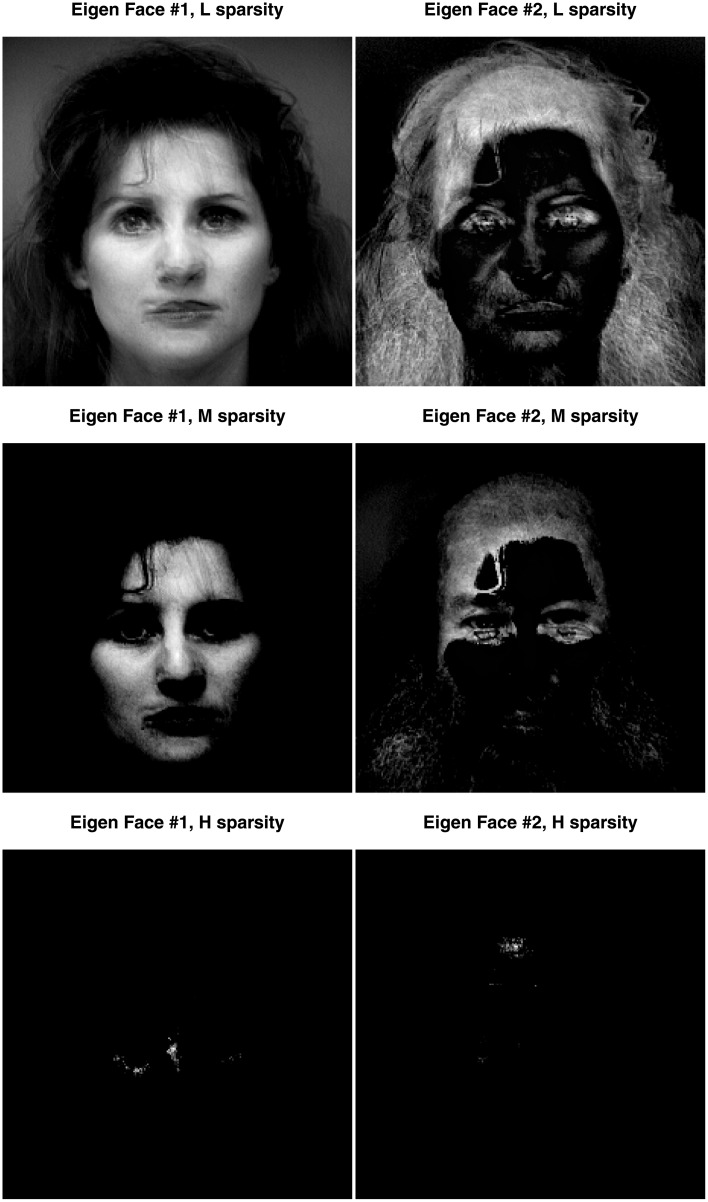
Face data. The pseudo-eigenfaces for Dimension 1 on the left column and Dimension 2 on the right column. For this graph, only the CSVD was applied, with three different levels of sparsity: low on the top row (L), medium on the middle row (M), and high on the bottom row (H).

**Fig 11:**
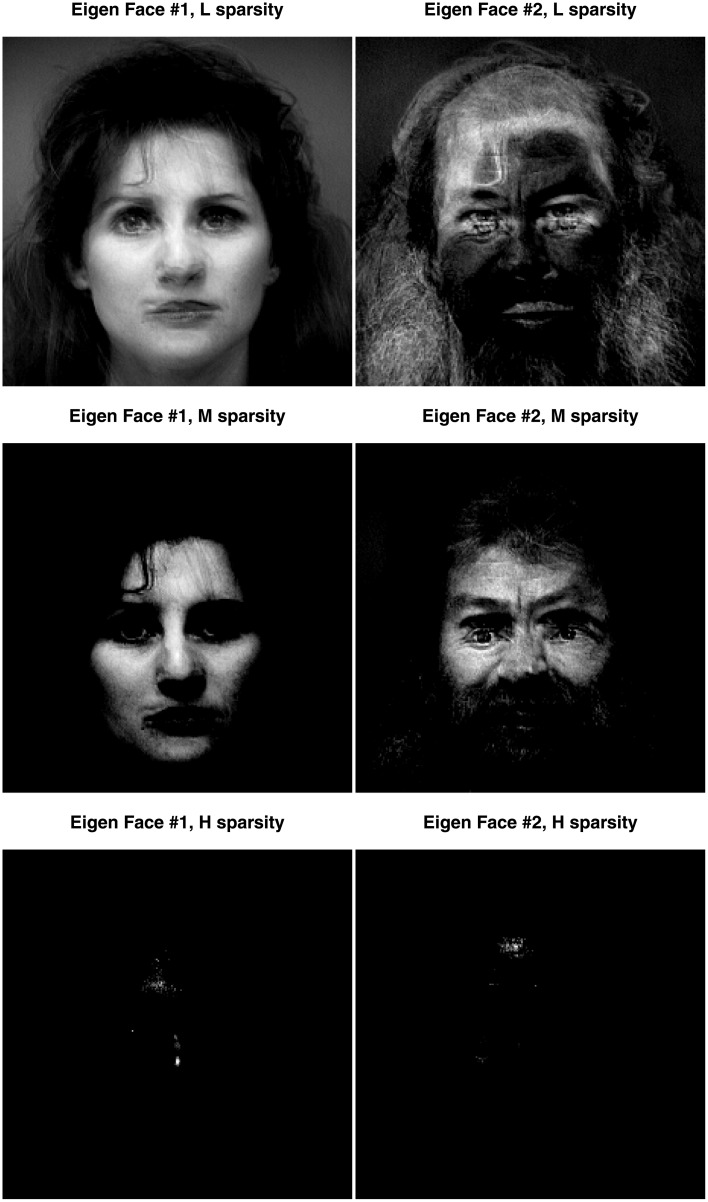
Face data. The pseudo-eigenfaces for Dimension 1 on the left column and Dimension 2 on the right column. For this graph, only PMD was applied, with three different levels of sparsity: low on the top row (L), medium on the middle row (M), and high on the bottom row (H).

**Fig 12:**
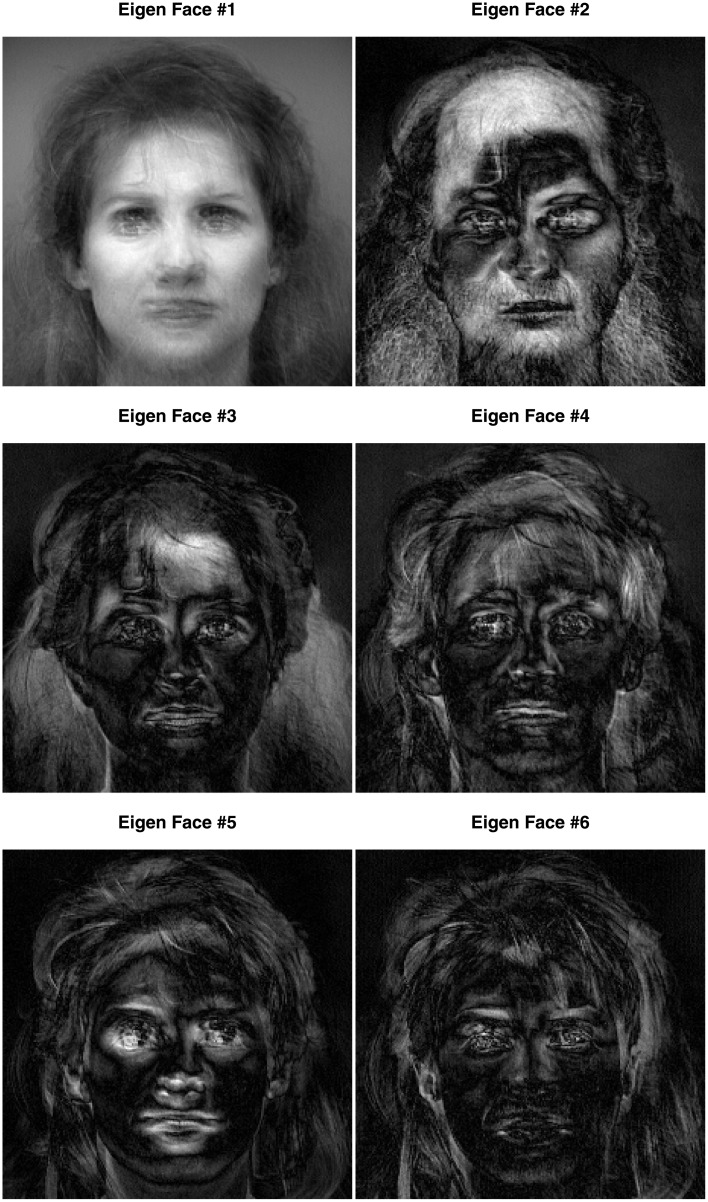
Face data. The six eigenfaces obtained from the plain SVD.

**Fig 13:**
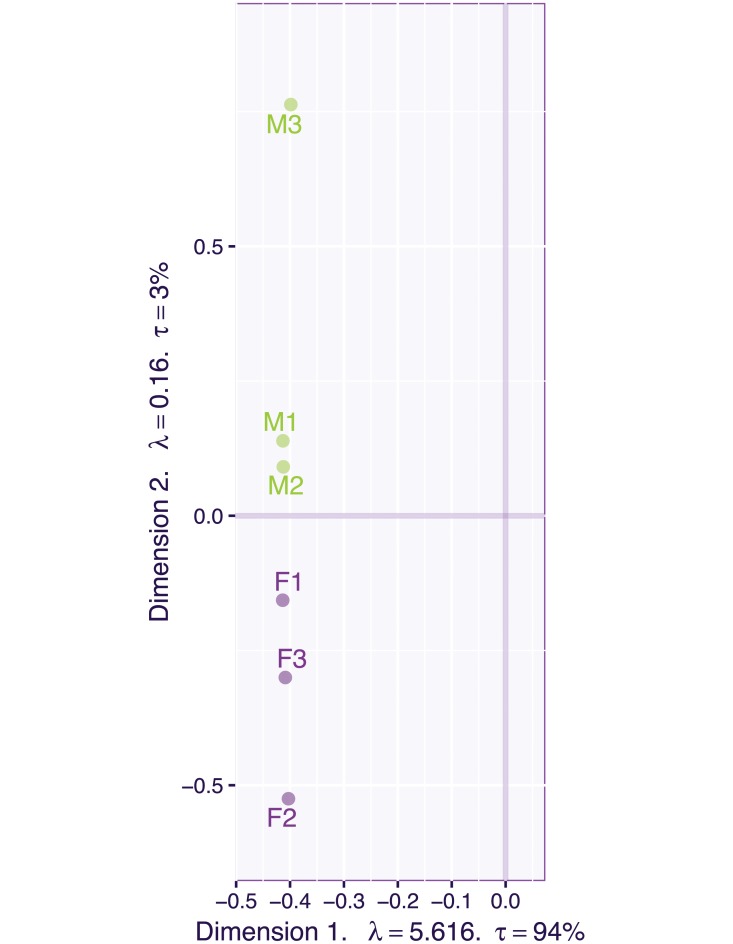
Face data. The two first left singular vectors of the plain SVD of the non-centered data.

We applied the CSVD and PMD to the face set using three different sparsity levels (low, medium, and high). Fig 9 shows the plot of the first two components for these three levels of sparsity. Both the CSVD and PMD tend to isolate the woman faces on the first dimension and the male faces on the second dimension. The corresponding first two eigen-faces, (see Figs 10 and 11) show that both the CSVD and PMD tend to extract characteristic features of the female faces (first eigen-face) or the male faces (second eigen-face). In contrast, the first and second eigen-faces for the plain SVD (plotted in Fig 12) show respectively a weighted average face (i.e., a linear combination with positive coefficients of the faces) and a mixture between male (with positive coefficients) and female (with negative coefficients) faces. This interpretation for the plain SVD is confirmed by Fig 13 where all faces load almost identically on Dimension 1, and where Dimension 2 separates men from women.

**Fig 14:**
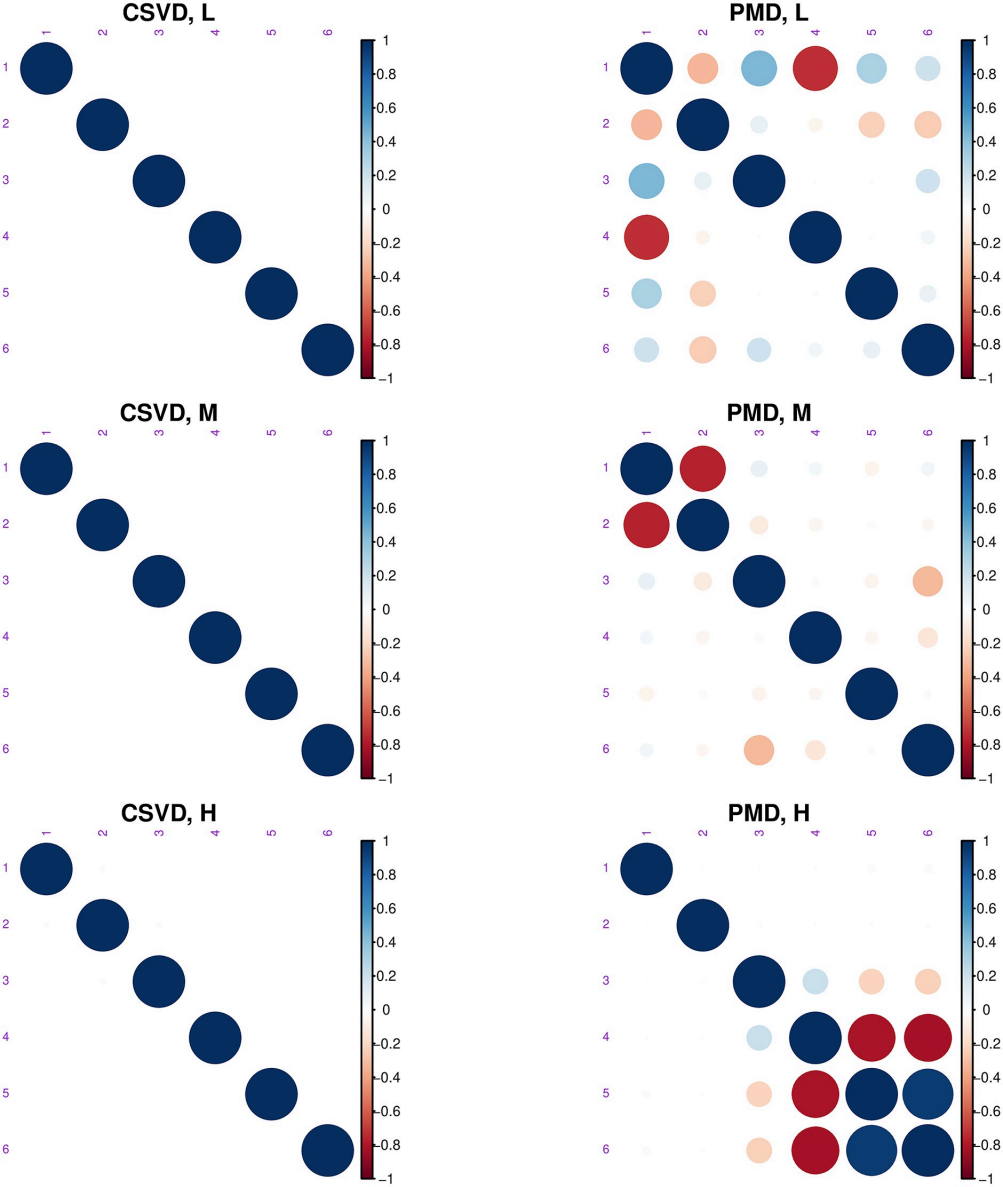
Face data. Cross-product matrix of the 6 right pseudo-singular vectors for three levels of sparsity: low (L), medium (M), and high (H). The results for the CSVD are reported on the left, and the results for PMD are reported on the right.

Overall the CSVD and PMD behave similarly and both show (compared to the plain SVD), that introducing sparsity can make the results easier to interpret because groups of individuals (men or women) can be identified and linked to small subset of variables (i.e., here pixels). However CSVD and PMD differ in the number of components suggested by their scree plot as indicated by Fig 7. The differences between the loadings estimated by both methods are also seen in Fig 14, which depicts the cross-product between the 6 right singular vectors for three different sparsity levels and for both methods. This figure shows that the risk of re-injecting variability, that was already described in previous components, increases with the sparsity parameter and the number of required components.

### 4.3 Psychometric example: The mental imagery questionnaire

These simulated data were created to match the loading structure of an original dataset obtained from 2,100 participants who—as part of a larger project on memory—answered an online version of the “object-spatial imagery questionnaire” (OSIQ, [29])—a psychometric instrument measuring mental imagery for objects and places. Using a 5-point rating scale, participants rated their agreement for 30 items (e.g., “I am a good Tetris player”) that should span a 2-dimensional space corresponding to the hypothesized spatial and object imagery psychometric factors.

The simulated data were obtained from an original data set by first performing a (centered and un-scaled) PCA on the original dataset and keeping only the loadings and the eigenvalues. Random pseudo-observations were generated by randomly sampling (with a uniform probability distribution) points in the factor space and then building back the corresponding data matrix from these random factor scores and the loadings. The final simulated data matrix was then obtained by scaling this new data matrix so that it only contains integer values whose distribution match, as best as possible, the original data matrix. This way, the simulated data matrix contains random values whose means, variances, and loadings roughly match the original data matrix. The R-code used to create the simulated data can be found from the R-package data4PCCAR (available from from the Github repository HerveAbdi/data4PCCAR; the simulated data matrix can also be found in the same R-package.

**Fig 15:**
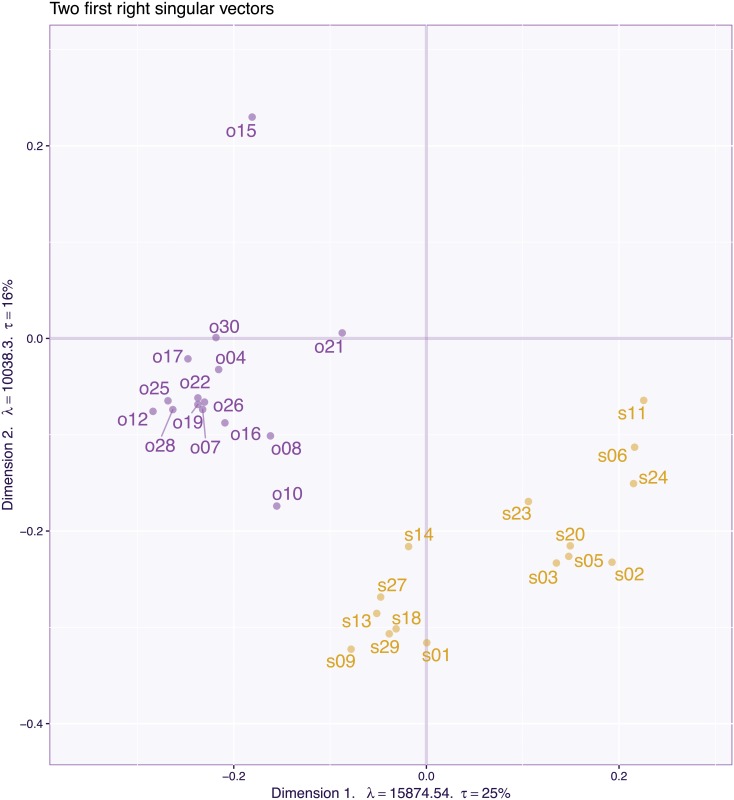
OSIQ data. Loadings of the first two principal components.

**Fig 16:**
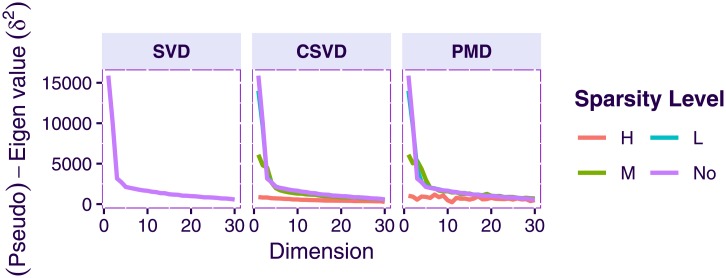
OSIQ data. Scree plots for SVD, the CSVD and PMD for different values of the sparsity parameter.

The 2,100 (participants) by 30 (items) data matrix was pre-processed by centering and normalizing each variable and was then analyzed by PCA (i.e., an SVD of the pre-processed matrix). Fig 15 plots the loadings for the 30 items for the first two components of the PCA. In this figure, each item is labeled by its number in the questionnaire (see [29] for details and list of questions), and its a priori category (i.e., “object” vs “spatial”) is indicated with the first letter (o vs s) and color (blue for “object” vs gold for “spatial”). The scree plot (see Fig 16 left) and the plot of the loadings for the first two dimensions (Fig 15) supports a two factor model (with the plane created by Dimensions 1 and 2 explaining 44% of the total variance). The pattern of the loadings, however, reveals that some items load, as predicted, on only one factor (most object items and some spatial items) but that roughly half of spatial items (i.e., s02, s03, s03, s05, s06, s11, s20, s23, s24) and, at least, one object item (i.e., o15) load on both Dimensions 1 and 2. These items are ambiguous because they can reflect either only one of the hypothesized factors or a combination of both factors. To simplify the interpretation, a standard psychometric approach would keep only the unambiguous items, re-run the analysis with these items, and “prettify” the solution with an orthogonal rotation such as Varimax [33].

To evaluate the effects of sparsification, we used three levels of sparsity (in addition to the “no sparsity” condition corresponding to the plain SVD): Low (L), Medium (M), and High (H). As expected, and illustrated by the scree plots (see Fig 16), sparsification reduced the amount of variance (i.e., the pseudo-eigenvalues) explained by the sparsified components.

**Fig 17:**
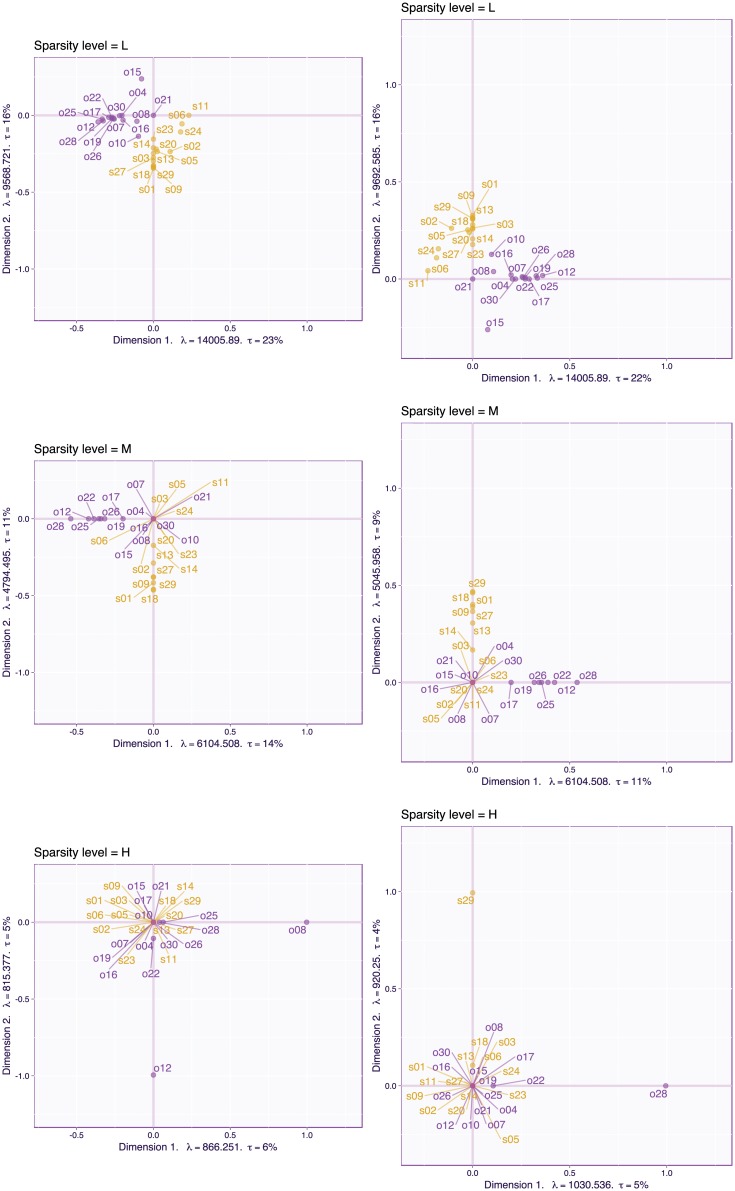
OSIQ data. Loadings of Dimensions 1 and 2 with an increasing degree of sparsity for both the CSVD (left column) and PMD (right column).

Fig 17 plots the item loadings for Dimensions 1 and 2 for both the CSVD (left column) and PMD (right column) as a function of the levels of sparsity (L/M/H). For the low and intermediate levels of sparsity. For the first two levels of sparsity (L and M) the CSVD and PMD give similar results, possibly because the factor structure of the items on the first dimension is strong enough to be recovered without the orthogonality constraints. For the highest level of sparsity, the CSVD and PMD single out the same item (o28) on the first dimension (an unsurprising result because the maximized criteria are equivalent) but single out different items (s29 vs s18) on the second dimension.

**Fig 18:**
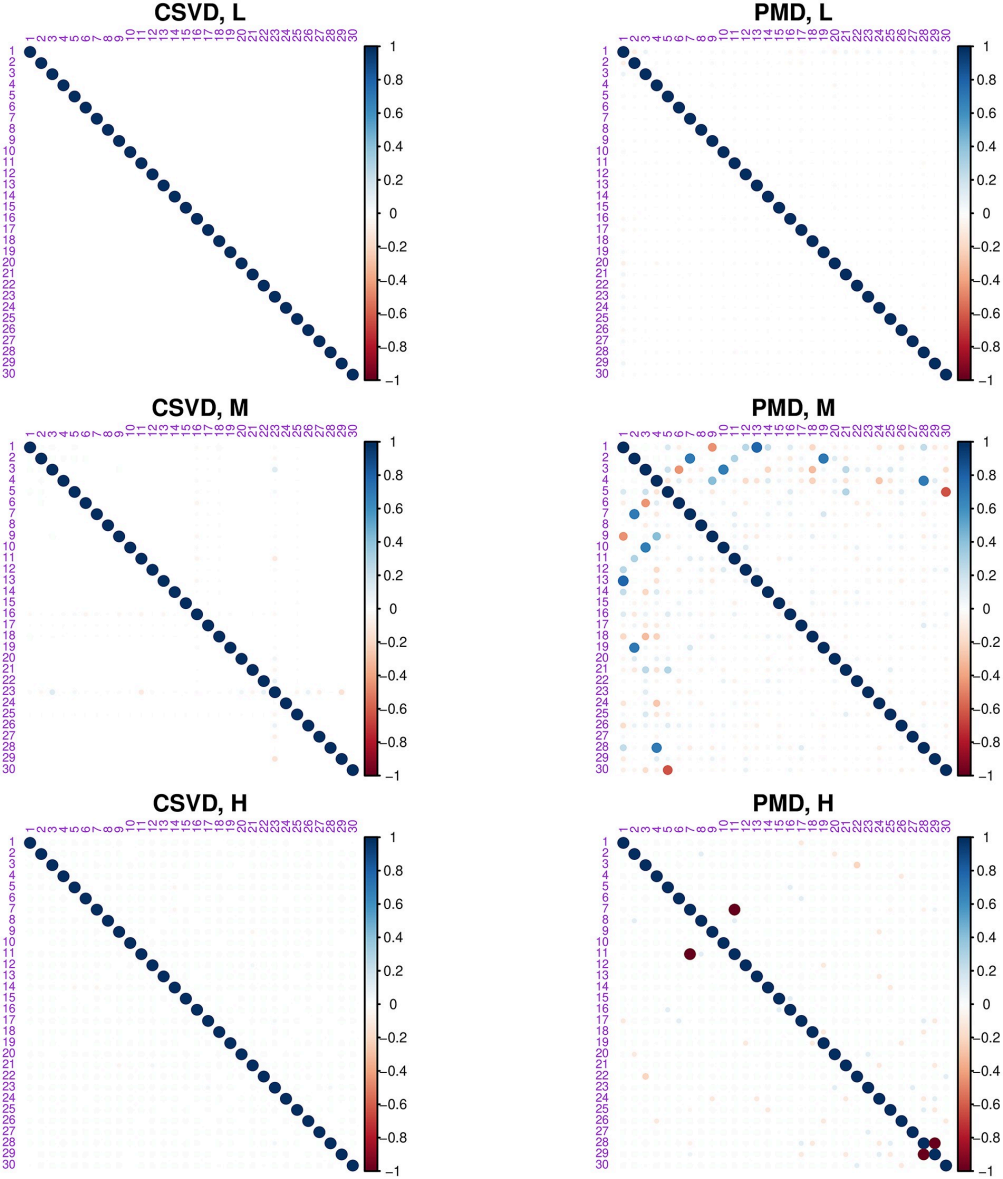
OSIQ data. Plot of the cross-product between the loadings obtained for up to 30 dimensions. Left: CSVD. Right: PMD. Top to bottom: the three different levels of sparsity.

Fig 18 plots the correlations between the loadings estimated with the CSVD or PMD for all 30 dimensions and shows again that the components extracted by PMD are correlated with other components.

**Fig 19:**
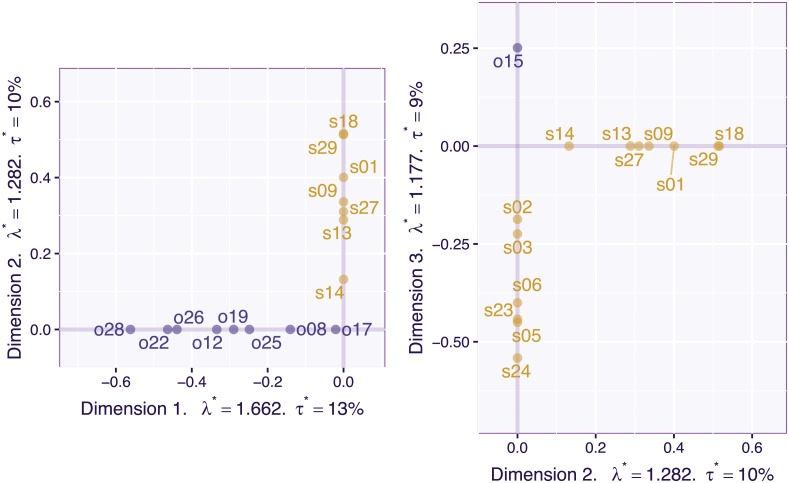
OSIQ data. Loadings for Dimensions 1, 2, and 3 with sparsity parameters set to *c*_1_ ≈ 15.11 and *c*_2_ ≈ 2.50. The sparsity parameters were empirically determined visually to create “pure” dimensions.

**Fig 20:**
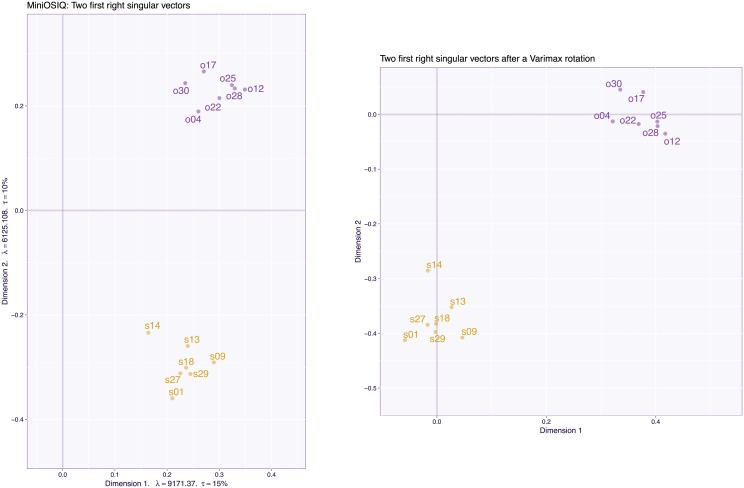
OSIQ data. PCA with the reduced set of 14 items from the OSIQ. Left: Dimensions 1 and 2 for plain PCA; Right: Dimensions 1 and 2 after a two-dimensional Varimax rotation.

Visual inspection of the plain PCA analysis suggests that the items are roughly clustered into three groups (pure object, pure spatial, and mixed spatial/objects). To better characterize these three groups of items, we ran an additional analysis in which we set the sparsity parameters to values (specifically $c_{1}\approx 0.55\sqrt{I=2,100}\approx 25.31$ and $c_{2}\approx 0.47\sqrt{J=30}\approx 2.56$ for, respectively the left and right singular vectors) that would generate three pure dimensions for the item loadings (see Fig 19). With this analysis, the first two dimensions isolate the pure items and the third dimension extracts the mixed items. To confirm this interpretation, we ran a plain PCA on the pure items (see Fig 20 left) followed by a Varimax rotation for two dimensions (see Fig 20 right). The Varimax rotated space recovered a solution equivalent to the CSVD with, however, the caveat, that Varimax required the a priori knowledge of the dimensionality of the space whereas the CSVD did not require this information.

## Discussion

The constrained singular value decomposition is a computationally efficient new method that sparsifies the SVD while preserving the orthogonality of the singular vectors. To do so, the CSVD expresses each constraint as a projection onto a convex set and integrates multiple constraints as the projection onto the intersection of the convex sets expressing the constraints (POCS). As shown in Appendix D, the CSVD algorithm is guaranteed to converge to a stable point because it is a member of the more general class of the *block successive upper-bound minimization (BSUM) algorithms*. The CSVD can easily be extended to incorporate additional constraints (e.g., group LASSO, metrics constraints of the rows or columns, spatial constraints) as long as these constraints can be expressed as projections onto convex sets.

To evaluate the relevance of the orthogonality constraints, we compared, on three examples, the plain SVD, the penalized matrix decomposition [11], and the CSVD. We found that, as could be expected, without the orthogonality constraint, higher singular vectors shared information with the earlier singular vectors—a problem likely to hinder the interpretation of these later components. The example using face images shows that the CSVD could extract, from the images, characteristic features defining clusters of observations (e.g., men vs women). The psychometric example illustrates how the CSVD can be used in lieu of rotation (e.g., Varimax) to identify psychometrically “pure” components without having to choose a priori the dimensionality of the space.

Of course, some questions remain open. For example, the choice of the sparsification constant is left to the user but this choice could be helped with some cross-validation schemes such as the one suggested by Witten et al. (see Algorithm 5 in [11]). In practice, this choice is likely to involve some trade-off between the interpretability of the pseudo singular vectors and the number of non-zeros loadings for a few of the first singular vectors. Along the same lines, and just like for the plain SVD, the number of (sparse) singular vectors to examine remains, in part, a subjective decision. Finally, the problem of the reliability of the sparsification, although a topic of great interest for sparse methods of prediction [34], remains open for sparse SVD or PCA and should be a topic for future research. Future directions should also include the integration of the CSVD into other methods that are traditionally based on the regular SVD—such as canonical correlation, or partial least squares correlation—or on the generalized SVD—such as correspondence analysis (see, e.g., [15] for previous relevant work along these lines).

The R package csvd implementing the constrained singular value decomposition is available for download from https://github.com/vguillemot/csvd.

## A The deflation operation generates orthogonal singular vectors

In this section, we show that repeatedly using the deflation operation will generate a set of left (respectively right) orthogonal singular vectors ordered by their singular value.

**Theorem 1** (Deflation). *The first singular triplet of the* (*k* + 1)*th deflated matrix is the* (*k* + 1)*th singular triplet of the original matrix*.

*Proof*. Assume that the *k* ≥ 1, singular values, left and right singular vectors of a given matrix **X** have been estimated and stored in matrices **Δ**_*k*_, **P**_*k*_ and **Q**_*k*_.

Let

$$\begin{array}{c} X^{\left(k+1\right)}=X-P_{k}\Delta_{k}Q_{k}^{\;\;⊤}, \end{array}$$ (21)

be the (*k* + 1)th deflated matrix with:

$$\begin{array}{c} P_{k}^{\;\;⊤}P_{k}=I\;\text{and}\;Q_{k}^{\;\;⊤}Q_{k}=I. \end{array}$$ (22)

Additionally, let *δ*, **p** and **q** be (respectively) the first singular value, left and right singular vectors of **X**^(*k*+1)^.

To prove Theorem 1, we show that *δ*, **p** and **q** are the (*k* + 1)th singular triplet of **X**.

First, because *δ*, **p** and **q** are a singular triplet of **X**^(*k*+1)^, we have:

$$\begin{array}{c} \delta q=X^{(k+1)⊤}p \end{array}$$ (23)

and

$$\begin{array}{c} \delta p=X^{\left(k+1\right)}q. \end{array}$$ (24)

Second, to prove that **q** is orthogonal to each column of **Q**_*k*_, we consider the quantity $Q_{k}^{\;\;⊤}q$. By multiplying both sides of Eq 23 by $Q_{k}^{\;\;⊤}$, developing, and simplifying we obtain

$$\begin{array}{cc} \delta Q_{k}^{\;\;⊤}q & =Q_{k}^{\;\;⊤}X^{(k+1)⊤}p\\ & =Q_{k}^{\;\;⊤}(X^{\;\;⊤}-Q_{k}\Delta_{k}P_{k}^{\;\;⊤})p\;(\text{Cf}.\;\text{Equation}\;21)\\ & =\underset{\Delta_{k}P_{k}^{\;\;⊤}}{\underset{︸}{Q_{k}^{\;\;⊤}X^{\;\;⊤}}}p-\underset{I}{\underset{︸}{Q_{k}^{\;\;⊤}Q_{k}}}\Delta_{k}P_{k}^{\;\;⊤}p\\ & =\Delta_{k}P_{k}^{\;\;⊤}p-\Delta_{k}P_{k}^{\;\;⊤}p\\ & =0. \end{array}$$ (25)

Therefore, when *δ* is not null, **q** is orthogonal to each column of **Q**_*k*_. A similar proof shows that **p** is orthogonal to each column of **P**_*k*_.

Third, because **p** (respectively, **q**) is orthogonal to each column of **P**_*k*_ (respectively **Q**_*k*_), we have

$$\begin{array}{c} X^{(k+1)⊤}p=\delta q,\text{and}\;X^{\left(k+1\right)}q=\delta p, \end{array}$$ (26)

which, combined with Eqs (23) and (24), implies that

$$\begin{array}{c} X^{\;\;⊤}p=\delta q,\text{and}\;Xq=\delta p, \end{array}$$ (27)

which, in turn, shows that *δ*, **p**, and **q** are a singular triplet of **X**. This proof also shows (*mutatis mutandis*) that any singular triplet of **X**^(*k*+1)^ is a singular triplet of **X**.

Finally, from the definition of **X**^(*k*+1)^, and because **X**^(*k*+1)^ is orthogonal to **P**_*k*_ **Δ**_*k*_ **Q**_*k*_, the rank of **X** is equal to the rank of **X**^(*k*+1)^ plus the rank of $P_{k}\Delta_{k}Q_{k}^{\;\;⊤}$, which, in run, implies that all the singular values of **X**^(*k*+1)^ are the remaining singular values of **X** and that the first singular value of **X**^(*k*+1)^ is the (*k* + 1)th singular value of **X**.

## B Only some values of the constraints lead to solutions

As stated by Witten et al. ([11], page 519 *ff*.) the constraint parameters *c*_1_ and *c*_2_ lead to solutions only when they are in the range:

$$\begin{array}{c} 1\leq c_{1}\leq \sqrt{I}\;\text{and}\;1\leq c_{2}\leq \sqrt{J}. \end{array}$$ (28)

Fig 1 in [11] describes the geometric intuition behind this range in $R^{2}$.

In this appendix, we provide a proof of Statement 28. First, we prove Lemma 1 (that directly implies Statement 28).

**Lemma 1**. Let $x\in R^{N}$, *then*

$$\begin{array}{c} \|x{\|}_{2}\leq \|x{\|}_{1}\leq \sqrt{N}\|x{\|}_{2} \end{array}$$ (29)

*Proof*. Assume that **x** belongs to $R^{N}$ and is different from **0**. The left side of the inequality is a consequence of Hölder’s inequality, which states that if 0 < *p* < +∞, and *q* is a positive real number such that $\frac{1}{p}+\frac{1}{q}=1$, then

$$\begin{array}{c} \|x{\|}_{2}^{2}\leq \|x{\|}_{p}\|x{\|}_{q}. \end{array}$$ (30)

With *p* = 1, this version of Hölder’s inequality becomes

$$\begin{array}{c} \|x{\|}_{2}^{2}\leq \|x{\|}_{1}\|x{\|}_{\infty }. \end{array}$$ (31)

Since ‖**x**‖_∞_ ≤ ‖**x**‖_2_, we have

$$\begin{array}{c} \|x{\|}_{2}\leq \|x{\|}_{1}. \end{array}$$ (32)

The right hand side of Eq 29 can be seen as a consequence of Cauchy-Schwarz inequality, which, in our case, would be formulated as follows:

$$\begin{array}{c} \forall a,b\in R^{N},|\langle a,b\rangle |\leq \|a{\|}_{2}\|b{\|}_{2}. \end{array}$$ (33)

If we set **a** to [|*x*_1_|, …, |*x*_*n*_|]^⊤^ and **b** to **1**, we obtain

$$\begin{array}{c} |\sum_{i=1}^{N}\left|x_{i}\right||\leq \|x{\|}_{2}\|1{\|}_{2}, \end{array}$$ (34)

which is equivalent to

$$\begin{array}{c} \|x{\|}_{1}\leq \sqrt{N}\|x{\|}_{2}. \end{array}$$ (35)

Putting together Eqs 32 and 35 gives:

$$\begin{array}{c} \|x{\|}_{2}\leq \|x{\|}_{1}\leq \sqrt{N}\|x{\|}_{2}\;. \end{array}$$ (36)

Lemma 1 implies that the constraints on the *L*_2_ and *L*_1_ norm of the left and right pseudo-singular vectors can be both active at the same time only if the sparsity parameter is chosen such that: (i) the *L*_1_-ball of radius *ρ* (i.e., $B_{L_{1}}\left(\rho \right)$) is entirely included in the *L*_2_-ball of radius 1 (i.e., $B_{L_{2}}\left(1\right)$) when *ρ* ≤ 1, and so that fulfilling the sparsity constraint implies that the *L*_2_ constraint is also fulfilled; and (ii) $B_{L_{2}}\left(1\right)$ is entirely included in $B_{L_{1}}\left(\rho \right)$ when $\rho \geq \sqrt{N}$, and so fulfilling the normalization constraint implies that the sparsity constraint is also fulfilled. To fulfill the constraints on both rows and columns of the CSVD gives the following range for the values of *c*_1_ and *c*_2_:

$$\begin{array}{c} 1\leq c_{1}\leq \sqrt{I}\;\;\text{and}\;\;1\leq c_{2}\leq \sqrt{J}, \end{array}$$ (37)

which proves the assertion.

## C A fast and exact algorithm for the projection onto the intersection of an *L*_1_ and *L*_2_ ball

In this section we describe a fast and exact algorithm for the projection onto the intersection of the *L*_1_-ball of radius *c* (i.e., $B_{L_{1}}\left(c\right)$) and the *L*_2_-ball of radius 1 (i.e., $B_{L_{2}}\left(1\right)$). This projection is defined by the following equation:

$$\begin{array}{c} \mathrm{proj}\left(x,B_{L_{1}}\left(c\right)\cap B_{L_{2}}\left(1\right)\right)=\{\begin{array}{c} \underset{y\in R^{N}}{\text{arg}\;\text{min}}\|y-x{\|}_{2}^{2},\\ \text{s}.\text{t}.\;y\in B_{L_{1}}\left(c\right)\cap B_{L_{2}}\left(1\right) \end{array} \end{array}$$ (38)

where $x\in R^{N}$, *N* is the number of variables of the dimension of interest (i.e., *I* or *J*), and *c* is the sparsity parameter (i.e., *c*_1_ or *c*_2_) with $1\leq c\leq \sqrt{N}$.

In [11], the solution of Eq 38 is computed using a binary search algorithm (BiSe). In the main part of our artcie, we propose to use the more general POCS algorithm. BiSe and POCS are iterative algorithms that give an approximate solution to Eq 38. In the case of the projection on the intersection of the *L*_1_ and *L*_2_ balls, the general POCS algorithm can be replaced by a fast and exact algorithm (see [26, 35]), that we call PL1L2 and detail in this appendix.

### Projection onto the *L*_1_-ball

The proposed approach implements an efficient algorithm for projecting a vector onto the *L*_1_-ball [35].

Let $\tilde{x}$ be the vector containing the absolute value of the components of **x** with its elements sorted in decreasing order. Additionally, we define the function *φ*(λ) = ‖*S*(**x**, λ)‖_1_. This function is continuous, piecewise linear and decreasing from $\varphi \left(0\right)=\|\tilde{x}{\|}_{1}$ to $\varphi ({\tilde{x}}_{1})=0$. Therefore, if ‖**x**‖_1_ ≥ *c*, since *φ* is continuous, there is a positive number λ such that *φ*(λ) = *c*. From this, we can deduce the algorithm of the projection onto the *L*_1_-ball of radius *c* that narrows down to 4 steps.

**Algorithm 6**: Fast projection onto the *L*_1_-ball.

**Data**: **x**, *c*

**Result**: ${\text{proj}}_{B_{L_{1}}\left(c\right)}\left(x\right)$

1. Take the absolute value of the components of **x** and sort them in decreasing order into a new vector $\tilde{x}$;

2. Find *i* such that $\varphi \left({\tilde{x}}_{i}\right)\leq c<\varphi \left({\tilde{x}}_{i+1}\right)$;

3. Find *δ* such that $\varphi ({\tilde{x}}_{i}-\delta )=c$. Since $\varphi \left({\tilde{x}}_{i}-\delta \right)=\sum_{k=1}^{i}{\tilde{x}}_{k}-i\left({\tilde{x}}_{i}-\delta \right)=\varphi \left({\tilde{x}}_{i}\right)+i\delta$, then $\delta =\frac{c-\varphi ({\tilde{x}}_{i})}{i}$;

4. Compute *S*(**x**, λ) with $\lambda ={\tilde{x}}_{i}-\delta$;

At the end of the algorithm, we obtain *S*(**x**, λ) which is now the projection of **x** onto $B_{L_{1}}\left(c\right)$. A similar algorithm was proposed in [36], [37], and [38].

### Projection onto the intersection of the *L*_1_ and *L*_2_-balls

In order to solve the optimization problem from Eq 38, we extend Algorithm 6 to the function

$$\begin{array}{c} \psi \left(\lambda \right)=\frac{\|S(\tilde{x},\lambda ){\|}_{1}}{\|S(\tilde{x},\lambda ){\|}_{2}}\;. \end{array}$$ (39)

We have the following Lemma:

**Lemma 2**. *Let* **x** *be a vector of* $R^{N}$, *composed of n* ≤ *N non-zero elements. Then*

$$\begin{array}{c} \|x{\|}_{1}\leq \sqrt{n}\|x{\|}_{2}\;. \end{array}$$ (40)

*Proof*. The proof of this Lemma is very similar to the proof given in Appendix B. Recall that as a consequence of the Cauchy-Schwarz inequality:

$$\begin{array}{c} \forall \;a,b\in R^{N},|\langle a,b\rangle |\leq \|a{\|}_{2}\|b{\|}_{2}. \end{array}$$ (41)

With **a** = [|*x*_1_|, …, |*x*_*N*_|] and **b** a vector such that

$$\begin{array}{c} b_{i}=\{\begin{array}{c} 1\;\text{if}\;x_{i}\neq 0,\\ 0\;\text{if}\;x_{i}=0, \end{array} \end{array}$$ (42)

the previous inequality becomes

$$\begin{array}{c} \sum_{i=1}^{N}b_{i}\left|x_{i}\right|\leq \|x{\|}_{2}\|b{\|}_{2}, \end{array}$$ (43)

which is equivalent to

$$\begin{array}{c} \|x{\|}_{1}\leq \sqrt{n}\|x{\|}_{2}. \end{array}$$ (44)

**Proposition 1**. For $\lambda \in [0;{\tilde{x}}_{1}[$,

$$\begin{array}{c} \psi \left(\lambda \right)=\frac{\|S(\tilde{x},\lambda ){\|}_{1}}{\|S(\tilde{x},\lambda ){\|}_{2}} \end{array}$$ (45)

*verifies the 3 following properties*:

1. *ψ is continuous and decreasing*.
2. *There exist an integer i and a positive real number δ, smaller than* ${\tilde{x}}_{i}-{\tilde{x}}_{i+1}$, *such that* $\psi ({\tilde{x}}_{i}-\delta )=c$.
3. *δ is the solution of a second degree polynomial equation*.

*Proof*. (**i**). The numerator and denominator of *ψ* are continuous because there are compositions of continuous functions. Moreover, for any λ strictly smaller than ${\tilde{x}}_{1}$, $\|S(\tilde{x},\lambda ){\|}_{2}\neq 0$. Therefore, *ψ* is continuous because it is the ratio of a continuous function and a non-zero continuous function.

Assuming ${\tilde{x}}_{N+1}=0$, for $\lambda \in [0;{\tilde{x}}_{1}[$ there exists *k* ∈ 1, …, *N* such that ${\tilde{x}}_{k+1}\leq \lambda <{\tilde{x}}_{k}$. For this specific λ, we have:

$$\begin{array}{c} \|S\left(\tilde{x},\lambda \right){\|}_{1}=(\sum_{j=1}^{k}{\tilde{x}}_{j})-k\lambda \end{array}$$ (46)

and

$$\begin{array}{c} \|S\left(\tilde{x},\lambda \right){\|}_{2}^{2}=\sum_{j=1}^{k}{\left({\tilde{x}}_{j}-\lambda \right)}^{2}=(\sum_{j=1}^{k}{\tilde{x}}_{j}^{2})-2\lambda (\sum_{j=1}^{k}{\tilde{x}}_{j})+k\lambda^{2}\;. \end{array}$$ (47)

Together, Eqs 46 and 47 imply that the derivative of *ψ* has the form:

$$\begin{array}{c} \psi^{\prime }\left(\lambda \right)=\frac{1}{\|S(\tilde{x},\lambda ){\|}_{2}^{2}}(\frac{\|S(\tilde{x},\lambda ){\|}_{1}^{2}}{\|S(\tilde{x},\lambda ){\|}_{2}}-k\|S(\tilde{x},\lambda ){\|}_{2})=\frac{1}{\|S(\tilde{x},\lambda ){\|}_{2}}\left(\psi {\left(\lambda \right)}^{2}-k\right)\;. \end{array}$$ (48)

Moreover, because the number of non-zero elements of vector $S(\tilde{x},\lambda )$ is equal to *k*, Lemma 2 implies that $\|S(\tilde{x},\lambda ){\|}_{1}\leq \sqrt{k}\|S(\tilde{x},\lambda ){\|}_{2}$, and therefore *ψ*(λ)^2^ ≤ *k*. As a consequence, *ψ*′(λ) ≤ 0, which, in turn, implies that *ψ*, being a continuous function with a negative derivative, is a decreasing function.

(**ii**). Let *N*_max_ be the number of elements of **x** equal to ${\tilde{x}}_{1}$ (the maximum of $\tilde{x}$) and $\nu \in [{\tilde{x}}_{2};{\tilde{x}}_{1}[$. Then

$$\begin{array}{c} \psi \left(\nu \right)=\frac{N_{\text{max}}\left({\tilde{x}}_{1}-\nu \right)}{\sqrt{N_{\text{max}}}\left({\tilde{x}}_{1}-\nu \right)}=\sqrt{N_{\text{max}}}. \end{array}$$ (49)

Thus, *ψ* is decreasing from $\psi \left(0\right)={\left\|x\right\|}_{1}/{\left\|x\right\|}_{2}\leq \sqrt{N}$ (Lemma 2) to $\psi \left(\nu \right)=\sqrt{N_{\text{max}}}$. This implies that for $c\in [\sqrt{N_{\text{max}}};\sqrt{N}]$, there is an integer *i* ∈ 1, …, *N* such that $\psi \left({\tilde{x}}_{i}\right)\leq c<\psi \left({\tilde{x}}_{i+1}\right)$. Finally, because *ψ* is continuous, there is a real number *δ* in $[0;{\tilde{x}}_{i}-{\tilde{x}}_{i+1}[$ such that $\psi ({\tilde{x}}_{i}-\delta )=c$.

(**iii**). Using the notations $\ell_{1}=\|S(\tilde{x},{\tilde{x}}_{i}){\|}_{1}$ and $\ell_{2}=\|S(\tilde{x},{\tilde{x}}_{i}){\|}_{2}$, with *i* (and *δ*) defined as previously stated in (**ii**), we have:

$$\begin{array}{cc} \|S(\tilde{x},{\tilde{x}}_{i}-\delta ){\|}_{1} & =\sum_{j=1}^{i}({\tilde{x}}_{j}-\left({\tilde{x}}_{i}-\delta \right))\\ & =\sum_{j=1}^{i}({\tilde{x}}_{j}-{\tilde{x}}_{i})+i\delta \\ & =\|S(\tilde{x},{\tilde{x}}_{i}){\|}_{1}+i\delta \\ & =\ell_{1}+i\delta \end{array}$$ (50)

and

$$\begin{array}{cc} \|S(\tilde{x},{\tilde{x}}_{i}-\delta ){\|}_{2}^{2} & =\sum_{j=1}^{i}{\left({\tilde{x}}_{j}-\left({\tilde{x}}_{i}-\delta \right)\right)}^{2}\\ & =\sum_{j=1}^{i}({\left({\tilde{x}}_{j}-{\tilde{x}}_{i}\right)}^{2}+2\delta \left({\tilde{x}}_{j}-{\tilde{x}}_{i}\right)+\delta^{2})\\ & =\ell_{2}^{2}+2\delta \ell_{1}+i\delta^{2}. \end{array}$$ (51)

Moreover, since

$$\begin{array}{c} \psi \left({\tilde{x}}_{i}-\delta \right)=c=\frac{\|S(\tilde{x},{\tilde{x}}_{i}-\delta ){\|}_{1}}{\|S(\tilde{x},{\tilde{x}}_{i}-\delta ){\|}_{2}}, \end{array}$$ (52)

the following equality holds:

$$\begin{array}{c} \|S(\tilde{x},{\tilde{x}}_{i}-\delta ){\|}_{1}^{2}=c^{2}\|S(\tilde{x},{\tilde{x}}_{i}-\delta ){\|}_{2}^{2}. \end{array}$$ (53)

Incorporating Eqs 50 and 51 into Eq 53 gives:

$$\begin{array}{c} \delta^{2}(i^{2}-ic^{2})+2\delta \ell_{1}(i-c^{2})+\ell_{1}^{2}-c^{2}\ell_{2}^{2}=0\;. \end{array}$$ (54)

The goal is now to find the positive root of this second degree polynomial equation. The discriminant Δ is equal to $4c^{2}\left(c^{2}-i\right)\left(\ell_{1}^{2}-i\ell_{2}^{2}\right)$. It remains to show that Δ is positive.

First, the number of non-zero elements of $S(\tilde{x},{\tilde{x}}_{i+1})$ is equal to *i* and Lemma 2 yields $\|S(\tilde{x},{\tilde{x}}_{i+1}){\|}_{1}\leq \sqrt{i}\|S(\tilde{x},{\tilde{x}}_{i+1}){\|}_{2}$. Second, $\psi \left({\tilde{x}}_{i+1}\right)=\frac{\|S(\tilde{x},{\tilde{x}}_{i+1}){\|}_{1}}{\|S(\tilde{x},{\tilde{x}}_{i+1}){\|}_{2}}>c$ so $\|S(\tilde{x},{\tilde{x}}_{i+1}){\|}_{1}>c\|S(\tilde{x},{\tilde{x}}_{i+1}){\|}_{2}$. Combining the two previous inequalities yields $\left(i-c^{2}\right){\left\|S\left(\tilde{x},{\tilde{x}}_{i+1}\right)\right\|}_{2}^{2}>0$ which implies that *i*−*c*^2^ > 0. Third, from $\psi \left({\tilde{x}}_{i}\right)=\ell_{1}/\ell_{2}\leq c<\sqrt{i}$, we deduce that $\ell_{1}^{2}-i\ell_{2}^{2}\leq 0$ which ensures that Δ is positive.

To conclude, the sign of $\frac{\ell_{1}^{2}-c^{2}\ell_{2}^{2}}{i^{2}-ic^{2}}$ corresponds to the sign of the product of the 2 roots. As this term is negative, the 2 roots have opposite signs. The single solution of $\psi ({\tilde{x}}_{i}-\delta )=c$ is:

$$\begin{array}{cc} \delta & =\frac{-2\ell_{1}\left(i-c^{2}\right)+\sqrt{\Delta }}{2i(i-c^{2})}\\ & =\frac{-2\ell_{1}\left(i-c^{2}\right)+2c\sqrt{\left[c^{2}-i\right]\left[\ell_{1}^{2}-i\ell_{2}^{2}\right]}}{2i(i-c^{2})}\\ & =-\frac{\ell_{1}}{i}+\frac{c}{i}\sqrt{\frac{i\ell_{2}^{2}-\ell_{1}^{2}}{i-c^{2}}}. \end{array}$$ (55)

Using the fact that $\psi \left({\tilde{x}}_{i}\right)=\ell_{1}/\ell_{2}$, the previous equation can be simplified as

$$\begin{array}{c} \delta =\frac{\|S(\tilde{x},{\tilde{x}}_{i}){\|}_{2}}{i}(c\sqrt{\frac{i-\psi {\left({\tilde{x}}_{i}\right)}^{2}}{i-c^{2}}}-\psi \left({\tilde{x}}_{i}\right)). \end{array}$$ (56)

We deduce from this a four step algorithm, called PL1L2, for the projection onto the intersection of the *L*_1_-ball of radius *c* and the *L*_2_-ball of radius 1.

**Algorithm 7**: PL1L2: an algorithm for a fast and exact projection onto $B_{L_{1}}\left(c\right)\cap B_{L_{2}}\left(1\right)$.

**Data**: **x**, *c*

**Result**: ${\text{proj}}_{B_{L_{1}}\left(c\right)\cap B_{L_{2}}\left(1\right)}\left(x\right)$

1. Take the absolute value of **x** and sort its elements in decreasing order to get $\tilde{x}$;

2. Find *i* such that $\psi \left({\tilde{x}}_{i+1}\right)\leq c<\psi \left({\tilde{x}}_{i}\right)$;

3. Let $\delta =\frac{\|S(\tilde{x},{\tilde{x}}_{i}){\|}_{2}}{i}(c\sqrt{\frac{i-\psi {\left({\tilde{x}}_{i}\right)}^{2}}{i-c^{2}}}-\psi \left({\tilde{x}}_{i}\right))$;

4. Compute *S*(**x**, λ) with $\lambda ={\tilde{x}}_{i}-\delta$;

## D Convergence of the CSVD algorithm

In this appendix we prove the convergence of the CSVD. To do so, we show that the CSVD is an instance of the *block successive upper-bound minimization (BSUM) algorithm* (introduced in [27]) and, as such, converges to a stationary point.

### D.1 Definitions and notations

Define *f*, which is the negative of the objective function from Eq 15

$$\begin{array}{c} f({[}_{q}^{p}])=-p^{\;\;⊤}Xq. \end{array}$$ (57)

The functions *u*_1_ and *u*_2_ are two “approximations” of *f*, defined as

$$\begin{array}{c} u_{1}(\tilde{p};{[}_{q}^{p}])=f({[}_{q}^{\tilde{p}}])=-{\tilde{p}}^{\;\;⊤}Xq \end{array}$$ (58)

and

$$\begin{array}{c} u_{2}(\tilde{q};{[}_{q}^{p}])=f({[}_{\tilde{q}}^{p}])=-p^{\;\;⊤}X\tilde{q} \end{array}$$ (59)

These two functions depend on the fixed given vectors **p** and **q** and vary according to $\tilde{p}\in R^{I}$ and $\tilde{q}\in R^{J}$.

In the BSUM framework, *f* is minimized by iteratively minimizing *u*_1_ over a convex set $P\subseteq R^{I}$ and *u*_2_ over a convex set $Q\subseteq R^{J}$.

**Definition 1** (BSUM algorithm [27]). *The BSUM algorithm (in the present setting) is defined as*:

1. *Minimize u*_1_ *over* P *with* **q** *fixed, and update* **p** *with the solution*;
2. *Minimize u*_2_ *over* Q *with* **p** *fixed, and update* **q** *with the solution*,

*and iterate until convergence*.

Recall the following definitions.

**Definition 2** (Directional derivative). *Let g be a function with gradient at* **x** *denoted* ∇_**x**_ *g*. *The directional derivative of g in a direction* **d** *is*

$$\begin{array}{c} g^{\prime }\left(x\;|\;d\right)=\left\langle \nabla_{x}g\;|\;d\right\rangle . \end{array}$$ (60)

**Definition 3** (Regularity). *Let f be a differentiable function defined over* P×Q. *Assume that*

$$\begin{array}{c} f^{\prime }({[}_{q}^{p}]|[\begin{array}{c} d_{1}\\ 0 \end{array}])\geq 0 \end{array}$$ (61)

*and*

$$\begin{array}{c} f^{\prime }({[}_{q}^{p}]|[\begin{array}{c} 0\\ d_{2} \end{array}])\geq 0 \end{array},$$ (62)

*with* $d_{1}\in R^{I}$ *and* $d_{2}\in R^{J}$. *If this implies that*

$$\begin{array}{c} f^{\prime }({[}_{q}^{p}]|[\begin{array}{c} d_{1}\\ d_{2} \end{array}])\geq 0 \end{array},$$ (63)

*then f is regular*.

### D.2 Equivalence of the BSUM algorithm and the CSVD

Algorithm 5 is equivalent to the BSUM algorithm because:

1. Minimizing *f* is the same as maximizing the objective function in Eq 15.
2. Minimizing *u*_1_ over P is equivalent to the left projection step.
3. Similarly, minimizing *u*_2_ is equivalent to the right projection step.

### D.3 Convergence of the BSUM algorithm

In order to converge to a stationary point, the BSUM algorithm needs to meet a few key assumptions that are specified in the following theorem (adapted from Theorem 2 in [27]).

**Theorem 2** (Convergence). *The BSUM algorithm converges to a stationary point under the following conditions*:

1. *f is regular*,
2. *u*_1_, *u*_2_ *and f coincide (condition (B1) in* [27]*)*
  $$\begin{array}{c} u_{1}(p;{[}_{q}^{p}])=u_{2}(q;{[}_{q}^{p}])=f({[}_{q}^{p}]),\;\forall p\in P,q\in Q \end{array}$$ (64)
3. *u*_1_, *u*_2_ *are upper bounds of f (condition (B2) in* [27]*)*, $\forall \tilde{p},p\in P$, *and* $\forall \tilde{q},q\in Q$
  $$\begin{array}{c} u_{1}(\tilde{p};{[}_{q}^{p}])\geq f({[}_{q}^{\tilde{p}}])\;and\;u_{2}(\tilde{q};{[}_{q}^{p}])\geq f({[}_{\tilde{q}}^{p}]), \end{array}$$ (65)
4. *the directional derivatives of u*_1_, *u*_2_ *and f coincide (condition (B3) in* [27]*)*
  $$\begin{array}{c} u_{1}^{\prime }(\tilde{p};{[}_{q}^{p}]|d_{1}){|}_{\tilde{p}=p}=f^{\prime }({[}_{q}^{p}]|[\begin{array}{c} d_{1}\\ 0 \end{array}]),s.t.p+d_{1}\in P, \end{array}$$ (66)
  *and*
  $$\begin{array}{c} u_{2}^{\prime }(\tilde{q};{[}_{q}^{p}]\left|d_{2}){|}_{\tilde{q}=p}=f^{\prime }({[}_{q}^{p}]\right|[\begin{array}{c} 0\\ d_{2} \end{array}]),s.t.q+d_{2}\in Q, \end{array}$$ (67)
5. *u*_1_ *and* *u*_2_ *are continuous functions (condition (B4) in* [27]*)*.

#### D.3.1 Regularity

We show in this section that *f* is regular. The gradient of *f* with respect to its arguments, **p** and **q**, is defined as

$$\begin{array}{c} \nabla f({[}_{q}^{p}])=[\begin{array}{c} -x_{q}\\ -x^{⊤}{}_{p} \end{array}]. \end{array}$$ (68)

Thus, the directional derivative of *f* in the direction $d={[}_{d_{2}}^{d_{1}}]$, with $d_{1}\in R^{I}$ and $d_{2}\in R^{J}$ is equal to

$$\begin{array}{c} f^{\prime }({[}_{q}^{p}]|d)=\nabla f{\left({[}_{q}^{p}]\right)}^{⊤}d=\underset{f^{\prime }({[}_{q}^{p}]|[\begin{array}{c} d_{1}\\ 0 \end{array}])}{\underset{︸}{-d_{1}^{⊤}Xq}}+\underset{f^{\prime }({[}_{q}^{p}]|[\begin{array}{c} 0\\ d_{2} \end{array}])}{\underset{︸}{-d_{2}^{⊤}X^{⊤}p}}. \end{array}$$ (69)

Hence, if $f^{\prime }({[}_{q}^{p}]|[\begin{array}{c} d_{1}\\ 0 \end{array}])\geq 0$ and $f^{\prime }({[}_{q}^{p}]|[\begin{array}{c} 0\\ d_{2} \end{array}])\geq 0$, then the directional derivative of *f* in the direction of **d** is also positive, which proves that *f* is regular.

#### D.3.2 (B1), (B2), and (B3) in Razaviyayn et al., 2013

Because *u*_1_ and *u*_2_ are equal to the function *f* with either **p** or **q** fixed, *u*_1_ and *u*_2_ coincide with *f*, which proves (B1). Necessarily, so do their directional derivatives, which proves (B3). Finally because they coincide, *u*_1_ and *u*_2_ are upper bounds of *f* [in the sense of the condition (B2) in [27]].

#### D.3.3 Continuity: (B4) in Razaviyayn et al., 2013

Being compositions of linear operations, the functions *u*_1_ and *u*_2_ are both continuous.

### D.4 Conclusion

It follows from these properties that the CSVD method, as described in Algorithm 5 being based on alternating between applying the projected power method with respect to **p** and to **q**, is a particular instance of the *block successive upper-bound minimization (BSUM) algorithm* ([27]). Therefore, any limit point of the CSVD method is a stationary point and so the CSVD converges to a stationary solution of Eq 15.

## Supporting information

S1 Table Simulated data. Additional results on broader settings.
(PDF)
