# Supplementary material for: A constrained singular value decomposition method that integrates sparsity and orthogonality
Source: PLoS One. 2019 Mar 13;14(3):e0211463. doi: 10.1371/journal.pone.0211463 (PMC6415851; doi:10.1371/journal.pone.0211463)
Supplement: S1 Table — Additional results on broader settings. (PDF) [file pone.0211463.s001.pdf]

Additional analyses on simulated data

Additional simulations were performed to better assess the performance of the CSVD and PMD relative to 1) the intensity of the signal to noise ratio and 2) the presence or absence of structure in (the covariance matrix of) the noise. Specifically, we explored the behavior of the plain SVD, the PMD, and the CSVD with: 1) low variance (i.e., .01) vs high variance (i.e., 1) noise, and 2) plain Gaussian noise vs correlated noise as defined by the noise being obtained from the product of a Gaussian noise matrix with the following Toeplitz matrix (only the lower triangular part is represented):

$$\begin{bmatrix} 1 & & & & \\ 1-\frac{0.9}{J-1} & 1 & & & \\ 1-2\frac{0.9}{J-1} & 1-\frac{0.9}{J-1} & \ddots & & \\ \vdots & \ddots & \ddots & \ddots & \\ 0.1 & \dots & 1-2\frac{0.9}{J-1} & 1-\frac{0.9}{J-1} & 1 \end{bmatrix}. \tag{1}$$

To evaluate the performance of the recovery of the ground truth, we used two indices. The first index, denoted $R^2_{\mathbf{P}}$ (respectively $R^2_{\mathbf{Q}}$) for the left (respectively right) singular vectors, measures the fit between the singular vectors of the original signal matrix (i.e., the ground truth) and the computed singular vectors. This index is computed by averaging the squared correlations of the first five estimated singular vectors with the ground truth as:

$$R^2_{\mathbf{P}} = \frac{1}{5}\sum \operatorname{diag}\left(\operatorname{cor}\left(\widehat{\mathbf{P}}, \mathbf{P}\right)\right)^2, \tag{2}$$

and

$$R^2_{\mathbf{Q}} = \frac{1}{5}\sum \operatorname{diag}\left(\operatorname{cor}\left(\widehat{\mathbf{Q}}, \mathbf{Q}\right)\right)^2, \tag{3}$$

where $\widehat{\mathbf{P}}$ and $\widehat{\mathbf{Q}}$ are the estimated matrices of the singular vectors.

The second index, denoted $D_{\mathbf{P}}$ (respectively $D_{\mathbf{Q}}$), measures the degree of orthogonality of the left (respectively right) singular vectors by computing the Euclidean distance between the identity matrix and the cross-product matrix of the left singular vectors $D_{\mathbf{P}}$ (respectively right singular vectors $D_{\mathbf{Q}}$) as:

$$D_{\mathbf{P}} = \left\|\widehat{\mathbf{P}}^\top\widehat{\mathbf{P}} - \mathbf{I}\right\|_F, \tag{4}$$

and

$$D_{\mathbf{Q}} = \left\|\widehat{\mathbf{Q}}^\top\widehat{\mathbf{Q}} - \mathbf{I}\right\|_F. \tag{5}$$

The results, displayed in Table 1, indicate that: 1) SVD, CSVD, and PMD perform equally poorly when the level of noise is high, and 2) that CSVD's ability to recover orthogonal singular vectors is unaffected by the amount of noise or the structure of the noise.

Table 1. Additional simulations.

Variance	Structure	Method	$R^2_{\mathbf{P}}$	$R^2_{\mathbf{Q}}$	$D_{\mathbf{P}}$	$D_{\mathbf{Q}}$
Low	No	SVD	1.000	1.000	~ 0	~ 0
High	No	SVD	0.027	0.015	~ 0	~ 0
Low	Yes	SVD	1.000	1.000	~ 0	~ 0
High	Yes	SVD	0.008	0.003	~ 0	~ 0
Low	No	CSVD	0.964	0.998	~ 0	~ 0
High	No	CSVD	0.031	0.011	~ 0	~ 0
Low	Yes	CSVD	0.964	0.998	~ 0	~ 0
High	Yes	CSVD	0.009	< 0.001	~ 0	~ 0
Low	No	PMD	0.967	0.998	0.086	3.993
High	No	PMD	0.032	0.013	0.055	0.048
Low	Yes	PMD	0.967	0.998	0.088	3.991
High	Yes	PMD	0.003	< 0.001	5.324	3.718
